# Acute Stress Modulates Social Approach and Social Maintenance in Adult Zebrafish

**DOI:** 10.1523/ENEURO.0491-22.2023

**Published:** 2023-09-08

**Authors:** Alexander Cook, Holger Beckmann, Rutkay Azap, Soojin Ryu

**Affiliations:** 1 Institute of Human Genetics, University Medical Center of Johannes Gutenberg University Mainz, 55116, Mainz, Germany; 2 Living Systems Institute, Faculty of Health and Life Sciences, University of Exeter, Exeter, EX4 4QD, United Kingdom; 3 Max Planck Institute for Medical Research, 69120, Heidelberg, Germany

**Keywords:** acute stress, social behavior, zebrafish

## Abstract

Stress alters social functioning in a complex manner. An important variable determining the final effects of stress is stressor intensity. However, the precise relationship between stressor intensity and social behavior is not well understood. Here, we investigate the effects of varying acute stressor intensity exposure on social behavior using adult zebrafish. We first establish a novel test using adult zebrafish that allows distinguishing fish’s drive to approach a social cue and its ability to engage and maintain social interaction within the same behavioral paradigm. Next, we combined this test with a new method to deliver an acute stress stimulus of varying intensities. Our results show that both social approach and social maintenance are reduced in adult zebrafish on acute stress exposure in an intensity-dependent manner. Interestingly, lower stress intensity reduces social maintenance without affecting the social approach, while a higher stress level is required to alter social approach. These results provide evidence for a direct correlation between acute stressor intensity and social functioning and suggest that distinct steps in social behavior are modulated differentially by the acute stress level.

## Visual Abstract

**Figure:**
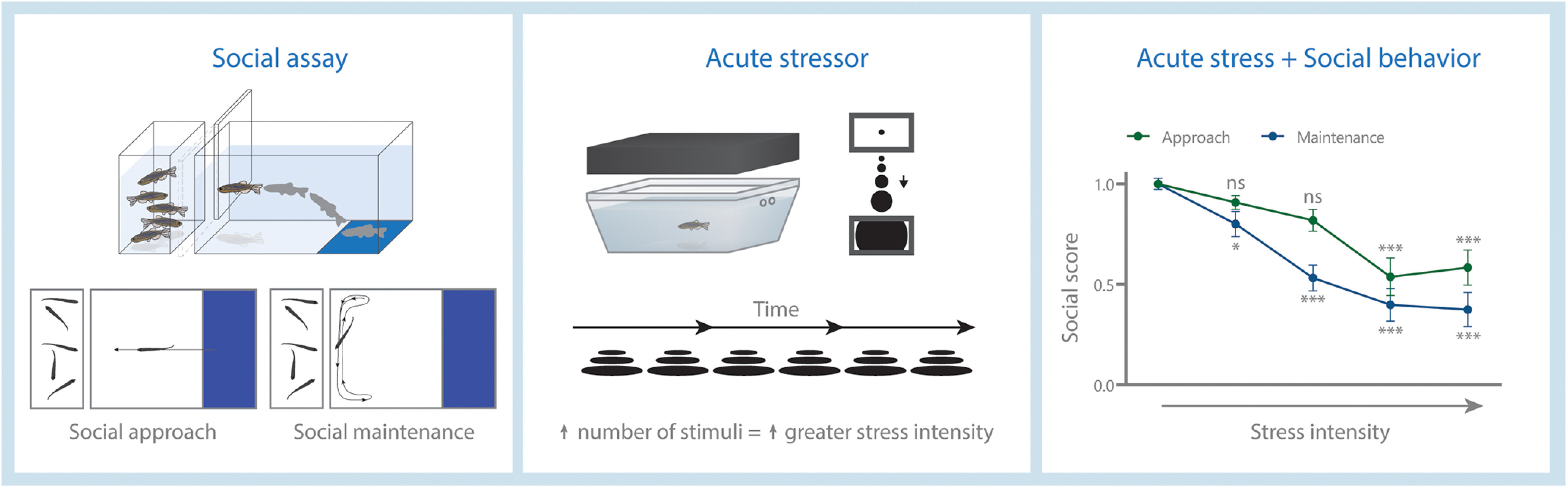

## Significance Statement

Acute stress exposure has a potent effect on social behavior in many animals including humans. However, so far, the effect of different stressor intensities on distinct steps of social behavior has not been directly tested. Here, using zebrafish, we develop a new social behavior paradigm and a new graded acute stressor delivery method to test the relationship between acute stressor intensity and social behavior. Our results show that acute stress modulates both social approach and social maintenance in an intensity-dependent manner but social maintenance is affected at lower stress intensity than social approach. Thus, this work reveals that distinct steps in social behavior are differentially modulated by the acute stress level.

## Introduction

Acute stress can profoundly impact social functioning of animals including humans (Sandi and Haller, 2015). However, the effects vary depending on the type of stressor, context, sex of the individual, specific tests employed, etc. (Beery and Kaufer, 2015; von Dawans et al., 2021). In rodents, studies suggest that acute stress exposure leads to reduced pro-social behaviors in terms of social motivation and interaction while increasing aggression (Anacker et al., 2016; Spencer, 2017). However, other studies demonstrate an increase in affiliative behavior and reduced aggression upon acute stress exposure (Wood et al., 2003; Muroy et al., 2016). In a striking example of varied stress effects, in prairie voles, the effects of acute stress on social preferences are sexually dimorphic (DeVries et al., 1995, 1996). An important variable determining the final effects of stress is the stressor intensity. Indeed, stress intensity and memory function follow an inverted U-shape in rodents (Salehi et al., 2010). Furthermore, the performance of innate behavior improves under moderate but not higher levels of acute stress in developing zebrafish larvae (Ryu and De Marco, 2017). However, the precise relationship between stressor intensity and social functioning is not well understood.

To determine the relationship between acute stressor intensity and social functioning, we use adult zebrafish in this study. Adult zebrafish show a high degree of sociality, where a range of social behaviors including aggression, shoaling, schooling, and mating have been well studied (R.F. Oliveira, 2013). Furthermore, the stress response systems of teleosts, including the hypothalamo-pituitary-interrenal (HPI) axis, show a high degree of similarity with those of mammals (Wendelaar Bonga, 1997). Social behavior in adult zebrafish is typically tested either with a social preference test using a single fish approaching conspecifics or by measuring shoaling parameters within a group of fish (Miller and Gerlai, 2012; Ogi et al., 2021). Some studies have examined the effect of acute stress on social behavior. For example, the presence of an aerial predator model or exposure to a novel environment resulted in altered group behavior, including higher shoal density in adult zebrafish (Miller and Gerlai, 2007; Kleinhappel et al., 2019). Using a social preference test, a reduction in social interaction can be induced by stress (Barcellos et al., 2020) or by pairing the social cue with an aversive stimulus (van Staden et al., 2020). However, the effect of varying stressor intensity on social behavior has not been studied so far in zebrafish.

Social functioning involves distinct behavioral sequences, broadly categorized as social recognition, social reward, and social maintenance (Chevallier et al., 2012). Several tests exist in rodents that measure different aspects of social behavior (Silverman et al., 2010). In contrast, current zebrafish social preference or shoaling tests do not allow for distinguishing different aspects of social behavior. Therefore, although social deficits in stressed fish have been reported (Abreu et al., 2016a; Giacomini et al., 2016; Barcellos et al., 2020), it is unclear which aspect of social functioning is affected. Therefore, a new behavioral paradigm is required to address this question. Typical methods to measure an animal’s motivation require animals to overcome a cost to gain access to reward (Dawkins, 1990; Kirkden and Pajor, 2006). Using such a principle, here we report, a novel behavioral paradigm that measures adult zebrafish’s drive to approach social cues and their ability to engage and maintain social interaction within the same experiment. We next combine this test with a new delivery method for graded acute stress exposure using a symbolic representation of an aerial-looming predator. Mimicking the aerial predator, a computer-generated looming dot (LD) presentation is used widely in combination with activity imaging to elicit robust escape behavior and to study fear-related circuits in larval zebrafish (Temizer et al., 2015). However, although used widely in larval zebrafish and in rodents, the use of looming dot (LD) in adult zebrafish has not been reported yet. Moreover, currently, there is no effective method to vary acute stressor intensity delivered to adult zebrafish. Therefore, we developed a suitable method for highly controllable and graded delivery of looming predator-related stimuli for adult fish. By combining the behavioral paradigm with graded acute stress delivery, we find that acute stress reduces both social approach and social maintenance in adult zebrafish in an intensity-dependent manner. Strikingly, we observed that social maintenance is affected by acute stressor exposure intensity that does not affect social approach. This indicates that distinct steps in social behavior may be modulated differentially by acute stress level and provides an entry point for dissecting underlying neural mechanisms.

## Materials and Methods

### Animal housing, husbandry, and experiments

Tübingen (TU) wild-type adult zebrafish (*Danio rerio*) bred in our facility were raised in mixed sex groups of 40 in 11-l tanks with a 12 h light (300–800 Lux, water surface)/12 h dark cycle. Fish were fed twice per day, once with artemia (Sanders, Great Salt Lake Artemia) and once with fish flakes (TetraMin Flakes). Water conditions were kept at 28 ± 0.5°C, 550 ± 50 μS conductivity, and 7.8 ± 0.3 pH. Between six- and nine-month-old females (unless stated otherwise; 300–600 mg of body weight) were taken from their home tanks and were moved into an experiment room one week before experimentation. In the experiment room, subjects were housed in groups of ten in 3-l tanks under the same housing conditions as the main housing facility. On the day of testing, fish were used in only one behavior test and all behavior experiments were conducted between 9 AM and 1 PM before feeding. Fifteen minutes after testing, some fish were killed in ice water for cortisol extraction. Zebrafish experimental procedures were conducted in compliance with the ethical guidelines of the German animal welfare law and approved by the local government (Landesuntersuchugsamt Rheinland-Pfalz, 23177-07/G20-1-033).

### Social behavior assay

The social behavioral assay setup is composed of a transparent cuboidal acrylic tank measuring 18 cm length (L) × 11 cm width (W) × 9 cm depth (D) filled up to 6 cm with fish housing water (1.2 l). The floor of the tank is covered with white vinyl (66%) and blue vinyl (33%). At the end of the white area of the tank, a white opaque acrylic divider is positioned in the 2 cm gap between the blue/white tank and the social cue tank and functions as a visual barrier. The social cue tank is a custom-made transparent cuboidal acrylic tank measuring 6 cm (L) × 13.5 cm (W) × 9 cm (D) filled up to 6 cm with fish housing water (0.7 l) and contains five conspecific wild-type female fish who were habituated in the tank for 30 min before testing. The two tanks were enclosed by white panels on two sides and an infrared back panel positioned behind the test area, which was used to generate contrast between the subject and the background. A photography dome covers the arena from external light and movement. A halo light provided homogeneous ambient light at 550 Lux (at the water surface level) and 3200 Kelvin. The assay was recorded using two cameras, one positioned above and one to the side of the tank (using an infrared filter) providing 3D *X*-, *Y*-, *Z*-location coordinates. An additional camera was positioned above the social cue tank to track their positions. Subjects were netted into the center of the tank and after 5 s, the recording started. After 60 s, the visual divider was manually moved to reveal the social cue. The subject was recorded for a further 120 s.

**Figure 1.**
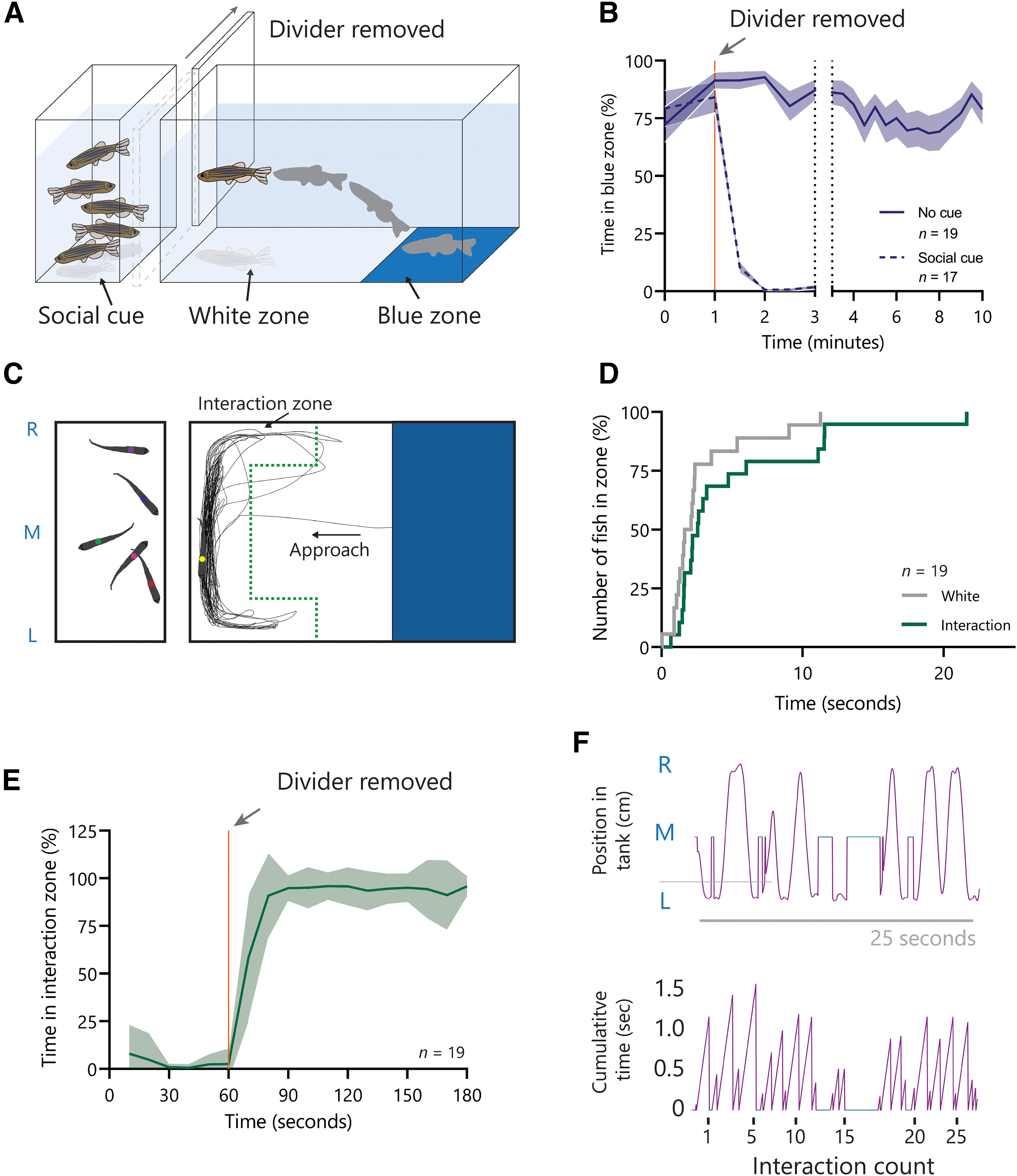
A novel social behavior assay distinguishes between social approach and social maintenance behaviors. ***A***, A 3D schematic representation of the social assay experimental setup with a divider obstructing the visual social cue. ***B***, Preference for the blue zone (%) with or without the divider blocking the visual access to social cues in 30-s bins. ***C***, A 2D schematic representation from the top view demonstrating typical traces of the test fish. The interaction zone is marked by the green dotted line. ***D***, A plot showing the time of the first entry of each fish to the white zone and subsequently to the interaction zone. Each event represents an individual’s first entry to each zone, displayed as a percentage of the population (%). ***E***, Time spent in the interaction zone with the divider (60 s) or without the divider (120 s) as a percentage (%) of 30-s bins. ***F***, The position of the test fish on the L-R axis during a typical 25-s period demonstrating stereotypical social interaction behavior. The cumulative time for each interaction is plotted demonstrating the typical duration and the frequency of interactions. Extended Data Figures 1-1, 1-2, and 1-3 are available to support this figure. Abbreviations: R, right; M, middle; L, left.

For analysis, the tank was divided into three zones: blue zone, white zone, and interaction zone (Fig. 1*A*,*C*). The interaction zone is the shape of a “C”; the size and shape of the zone were calculated to encompass 95% of the activity of a wild-type control fish. The time spent in each zone was calculated as the percentage of the presocial phase (first 60 s, no social cue visible) and the social phase (latter 120 s, with social cue visible) that was spent in each zone. Latency to enter a zone was measured as the initial entry to a zone following the beginning of the assay or removal of the divider (dependent on the phase stated). Interactions were counted as lateral movements in one direction while in the interaction zone, a new interaction started if the subject changed direction or left and reentered the interaction zone.

Social behavior is divided into two constructs, social approach and social maintenance. Social approach is the average normalized score for the time spent in the white zone (%) and the latency to approach the interaction zone (s). Social maintenance is an average normalized score for the time spent in the interaction zone (%) and the number of interactions performed. To normalize the data, first, the average readout for each measure was calculated for the “basal” group, giving the “basal average.” Each individual point was then divided by the basal average to give a “normalized readout,” with 1 being equal to the basal average. Next, the average normalized readout for time in the white zone and latency to approach the interaction zone was calculated to give an approach score for each fish [(time in the white zone/latency to approach the interaction zone)/2]. The same calculation was performed for social maintenance using the time spent in the interaction zone and the number of interactions [(time spent in the interaction zone/number of interactions)/2].

### Looming dot acute stress stimulus

A single fish was netted from their home tank in the experiment room to a smaller tank 16.9–18.7 cm (L) × 8.7 cm (W) × 10 cm height (H) of which three sides and the floor are sanded to reduce reflections. The tank was immediately placed underneath a Raspberry Pi display (Raspberry Pi 3 7” display 15.4 cm × 8.6 cm), mounted 2 cm from the top of the tank. Each subject was given 60 s to habituate to the tank before the recording began. Recording (Basler GenICam 2) and live-tracking (EthoVision XT 13, Noldus Information Technology) were performed at 30 FPS and a resolution of 1280 × 960 during the trial. After 30 s of recording baseline behavior (pre-LD phase), the LD stimulus was presented on the display.

The display was controlled by a Raspberry Pi (Model B) running a Linux operating system controlled via a custom-built graphical user interface (GUI) on the master PC to generate and present graphics interchange format (GIF) images. The custom-built GUI control panel generates a short sequence, which contains a list of 15 parameters for the programs on the Raspberry PIs. This sequence can be printed as a txt file on the Raspberry PIs via WLAN (PLINK). Custom-built scripts read the txt file, which was printed by the control panel on the Raspberry PI and the visualization process starts and creates the background in the right color and the expanding dots. The looming dot (LD) stimulus was an expanding black circle on a white background that started at the center point and filled most of the display. Grayscale was initially tested as a parameter for stress intensity, by changing the intensity of the dot from 25%, 50%, 75%, and 100% and presenting 12 dots. From the data 100% intensity was chosen and next the number of dots shown was 0, 6, 12, or 18 depending on the treatment group. The color of the dot was black on a white background, and each dot expanded for 2.8 s. After reaching the maximum size, the dot covered the screen for 1 s, after which a white background was presented for 1 s before the next dot was presented. Each LD event lasted 4.8 s, which means that the duration of the LD phase was equal to the number of LD × 4.8 s. Following the final LD presentation, the fish were recorded for a further 60 s (recovery phase).

*X*- and *Y*-coordinates were generated from the live tracking and the data were processed in Python to generate behavior parameter data which was graphed and statistically analyzed in Prism 9 (GraphPad Software). The average speed was calculated as the distance moved per second based on *X*- and *Y*-coordinates for the duration of each phase. Excess distance was the cumulative distance swum during the LD phase minus the distance swum by the 0 LD group. Turn frequency was calculated as the number of times the swimming direction changed on the *x*-axis per second for the duration of each phase. When combined with the social assay, fish were first subjected to the LD protocol with the desired number of LD and immediately following the recovery phase they were netted from the LD treatment tank to the social assay tank.

### Color preference test

Four tanks 24 cm (L) × 13.5 cm (W) × 130 cm (D) were created with 50% of the base of the tank covered with blue, green, red, or yellow and the other half white (same material and color as the social assay). The tanks were filled with 1.25 l of system water. On the day of testing, fish were netted into the tank from their home tank and recorded for 5 min. Five fish were tested with white on the left and different colors on the right and five fish were tested with the tank colors flipped to account for any biases created by other variables. The time spent in the colored zone was measured over the entire trial and presented as a percentage of the 5-min trial. A χ^2^ test was performed for each tank variation for statistical significance.

### Analysis of behavior

Behavior was tracked using EthoVision XT 13, Noldus Information Technology live tracking. Centroid *X*-, *Y*-, and *Z*-coordinates (only *X* and *Y* for LD assay) scaled to cm for each frame (30 FPS) were imported into python and converted to “pandas” data frames. For the social assay, Y coordinates from the top camera were converted to *Z*-coordinates and were merged with the *X*- and *Y*-coordinates of the side view camera. Data from each experimental trial were concatenated and labeled by experiment date, treatment group, and fish ID number. Based on *X*-, *Y*-, and *Z*-coordinates and the frame number, the distance, direction, and spatial metrics were calculated. Speed was calculated as distance moved (cm) per frame (cm per frame) and adjusted to distance per second [cm per second = (cm per frame × 30)]. Freezing duration was calculated as the number of frames under the freezing threshold. The freezing threshold was set at 10 consecutive frames where <0.1 cm was moved. Turns were calculated as changes in direction frame-by-frame using only *X*- and *Z*-coordinates in the social assay and *X*- and *Y*-coordinates in the LD assay. Information on zone preference and entry was calculated by assigning each frame to a zone based on coordinates.

For analysis of the movement of conspecifics, videos were generated using a top view camera. Videos were imported into python and using a script inspired by and using tools from Traktor (Sridhar et al., 2019). Centroid *X*- and *Y*-coordinates were generated for each conspecific, however the individual ID of each fish was not maintained. The nearest fish distance (distance on *z*-axis between test fish and nearest conspecific fish) was calculated based on the coordinates of the conspecific fish closest to the test fish on the *z*-axis that was within 1 cm of the dividing glass.

The covariance matrix was generated in python using the StandardScaler and decomposition. PCA tools by scikit-learn and plotted using the heatmap tool by seaborn.

### Diazepam treatment

Subjects treated with diazepam were housed in groups of 10 in their home tank, which was disconnected from the circulating water system in the experiment room. Diazepam (Diazepam-ratiopharm, PZN: 02232507) mixed in 10 ml of distilled water was added to the tank to reach the desired concentration (156 nm) in a volume of 1.5 l. After 2 h of Diazepam treatment, subjects were placed into the LD stimulus tank for testing.

### Whole-body cortisol extraction and assay

After LD exposure, fish were allowed to rest for 15 min before being netted into ice water (2°C) for 30 s and then they were immediately placed into a 5-ml tube and submerged into dry ice and ethanol for 10 min and finally frozen at −80°C until further processing. For cortisol extraction, 1 ml/100 mg (fish weight) of distilled water was added to the tube and the fish were thawed at room temperature. The fish were homogenized (25K, IKA disperser) for 60 s or until it was possible to pipette the homogenate. 1 ml of homogenate was moved into a 1.5 ml Eppendorf tube and centrifuged (10,000 rpm, 5 min, 4°C). The supernatant was added to an Eppendorf tube containing ethyl acetate 99.5% and vortexed for 60 s. The tube was centrifuged (5000 rpm, 5 min, 4°C) again and then placed into a −80°C freezer for 10 min. The ethyl acetate (supernatant) was transferred to a new tube and was evaporated (Eppendorf, Concentrator 5301, 30°C). The pellet was then resuspended in 20 μl of diluent. The samples then entered a competitive cortisol assay (Cisbio HTRF Cortisol kit, 62CRTPEG) and the plate was read (TECAN, infinite M1000 PRO). The data are calculated and presented as cortisol (μg) per weight of fish (g).

### Experimental design and statistical analysis

Group differences were analyzed using GraphPad Prism 9. The *n* for each group is reported in the figure or figure legend. The variance in data for each group is represented by the SEM and individual data points are plotted when necessary. No outliers were purposefully removed during conducting the experiment or in analysis. Variations in *n* are a result of data lost during acquisition or experimental design. Control groups and certain treatment groups may have more *n* in certain experiments as baseline behaviors and effects were assessed before entire datasets were generated to reduce the number of animals used. In cases where a figure includes multiple experiment days, each group was tested on every experimental day and day-to-day variations were assessed and no significant differences were found.

The statistical test used for each comparison is reported in Results. The tests were selected based on the number of comparisons and distribution of the data. For comparison of two groups either an unpaired *t* test (if data are Gaussian) or a Mann–Whitney (non-Gaussian data) test was used based on the distribution of data. In the case of the groups being the same individuals from different time points, a paired *t* test was selected. For comparisons of more than two groups (data are Gaussian) under the effect of one independent variable, a one-way ANOVA was used followed by either a Tukey’s *post hoc* analysis for comparing all possible group combinations or a Dunnett’s test for comparing all groups against one control group. For the comparison of more than two groups (data are not Gaussian) and the effect of one independent variable, a Kruskall–Wallis test was employed followed by a Dunn’s *post hoc* test to compare groups. For assessing the effects of two independent variables (e.g., phase X treatment), a two-way ANOVA (data are Gaussian) test was selected. For multiple comparisons analysis following a two-way ANOVA, a Šídák’s multiple comparisons *post hoc* analysis was used. Correlation is presented as the *R*^2^ value generated using simple linear regression analysis using GraphPad Prism; *p*-value adjustments were set as the default method for each multiple comparisons test in GraphPad Prism; *p*-values presented at 95% confidence levels with an α level of 0.05; ns *p* ≥ 0.05; *p < 0.05, **p < 0.01, ***p < 0.001.

## Results

### A novel assay affords distinguishing a drive for social approach and social maintenance

To measure a drive for social interaction, we sought to create a conflict between an unfavorable cue and rewarding social stimuli, where fish have to enter an unfavorable area to gain access to the reward. The test fish was introduced into the main tank that has two colored zones, blue and white. Zebrafish exhibit color preference and it has been previously demonstrated that blue is preferred (Bault et al., 2015; J. Oliveira et al., 2015; Park et al., 2016). The white zone is considered unfavorable in our assay following results demonstrating that the white zone was avoided compared with blue, green, red, and yellow zones in the color preference test. A χ^2^ test found that this preference was significant for each color, blue (*p* > 0.001), green (*p* > 0.001), red (*p* > 0.001), and yellow (*p* = 0.0164; Extended Data Fig. 1-1). There was no zone bias with a neutral floor of the tank (Extended Data Fig. 1-2). The main tank was separated from another tank holding five conspecific fish, which only become visible when a divider positioned between these two tanks is removed (Fig. 1*A*). When placed in a tank with blue and white-colored areas and with the divider in place, 89.2% of fish entered the blue zone in the first 10 s, after being netted into the center of the tank. The blue preference and white avoidance were maintained for a minimum of 10 min, during which period, the average time in the blue zone did not fall below 69% (Fig. 1*B*). When the divider was removed after 60 s, revealing the social cue, fish left the blue zone and entered the white zone, which they previously avoided. For the initial 60 s with the divider in place, fish spent on average 18.3% of their time in the white zone. This increased to 96.6% for the latter 120 s of the trial when the social cue was visible (Fig. 1*B*). After entering the white zone, fish approached the social cue directly. The fish swam as close to the wall as possible and exhibited repetitive lateral movements along the left-right (L-R) axis followed by sharp turns to come back to the wall separating it from its conspecifics. This stereotypical movement resulted in a C-shaped zone within the white zone, which we term the interaction zone (Fig. 1*C*). The average time that test fish took from first leaving the blue zone, traversing the white zone and entering the interaction zone was 2.21 s, indicating that fish left the blue zone to directly approach the social cue. For 94.7% of fish, this action was initiated within 12 s from the point when the divider had been removed (Fig. 1*D*). During the 120 s period after removing the divider, fish spent an average of 91.26% of their time in the interaction zone in close proximity to the social cue (Fig. 1*E*). We used females for all our behavioral tests in this study. However, the time in the interaction zone over 120 s was similar between female and male test fish when tested against five female social cue fish (Extended Data Fig. 1-3). Within the interaction zone, we noted that fish made stereotypical and repetitive L-R lateral movements. We defined each of these lateral movements as one interaction regardless of the direction. One interaction can be left to right or right to left. On average a fish performed interactions at a frequency of about one interaction per second, which was consistent across the 120-s trial period (Fig. 1*F*).

10.1523/ENEURO.0491-22.2023.f1-1 Extended Data Figure 1-1 White is least preferable compared to other colors. Time spent in the colored zone (blue, green, red, or yellow) compared to the white zone over a 5-min trial presented as a percentage of the trial duration (*p <* *0.05, ***p <* *0.001). Download Figure 1-1, TIF file.

**Figure 2.**
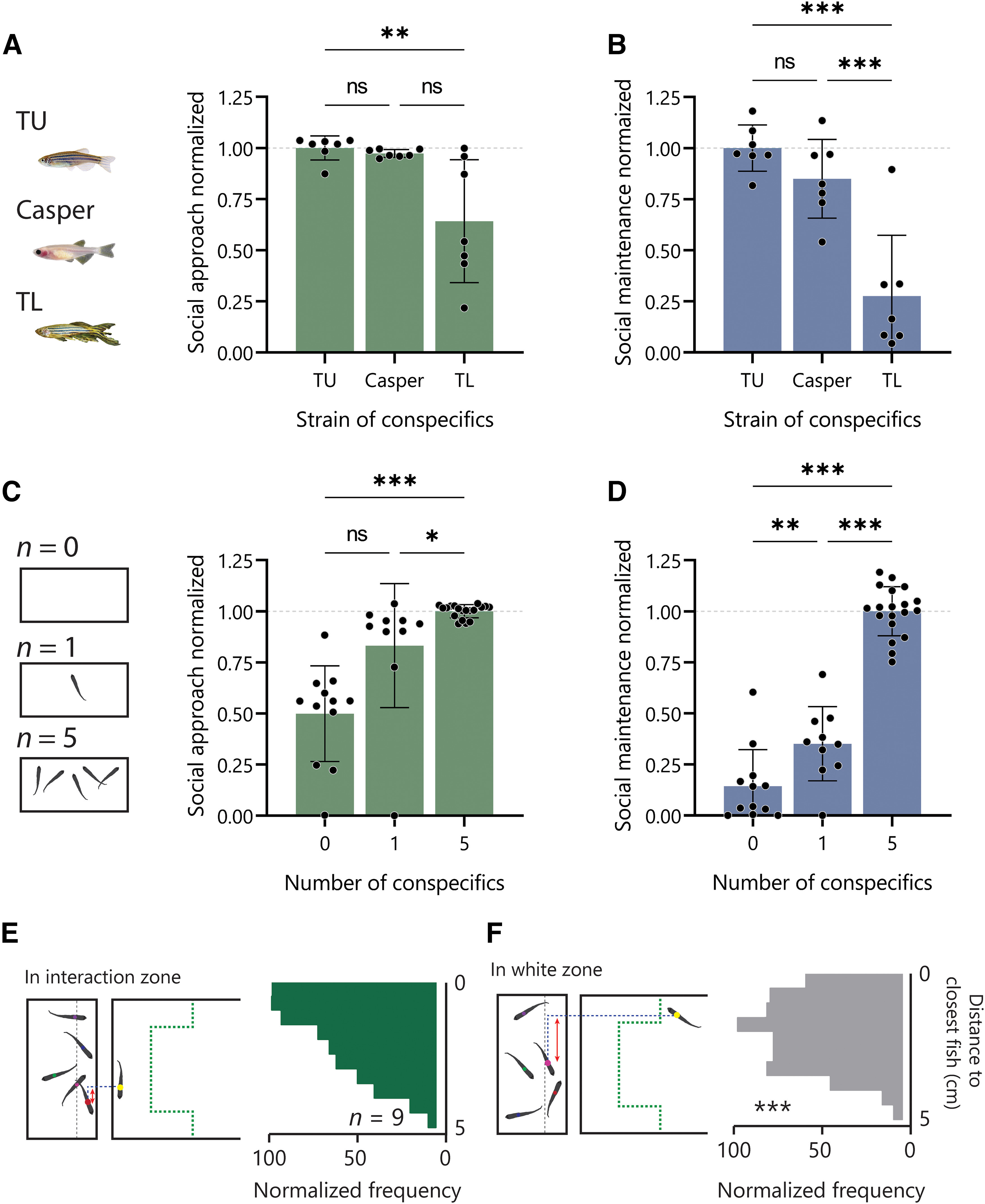
Social approach and social maintenance behaviors are modulated by the salience of the social cue. ***A***, Social approach behavior (normalized white zone preference and interaction zone latency) with TU (same as test fish), Casper, or TL fish as the social cue. ***B***, Social maintenance behavior (normalized time in the interaction zone and interaction count) with different strains as conspecifics. ***C***, Social approach behavior with zero, one, or five fish as the social cue. ***D***, Social maintenance behavior with different numbers of fish as the social cue. ***E***, The distance to the nearest fish is measured between the test fish and the nearest fish on the L-R axis that is within the 1-cm proximity zone (gray vertical dashed line). The distance to the nearest fish is plotted in histograms for when the test fish is in the interaction zone and when it is not in the interaction zone. ***F***, The distance to the nearest fish on the L-R axis plotted in a histogram for when the test fish is not in the interaction zone. Error bars denote the mean ± 1 standard deviation (SD); significant post hoc comparisons, ns *P* > 0.05; *P < 0.05, **P < 0.01; ***P < 0.001. Abbreviations: TU, Tübingen; TL, Tupfel Long-fin.

10.1523/ENEURO.0491-22.2023.f1-2 Extended Data Figure 1-2 There is no area bias in a neutral tank. Time spent in four equally divided zones 1, 2, 3, and 4 displayed as a percentage of the 5-min trial time. Download Figure 1-2, TIF file.

**Figure 3.**
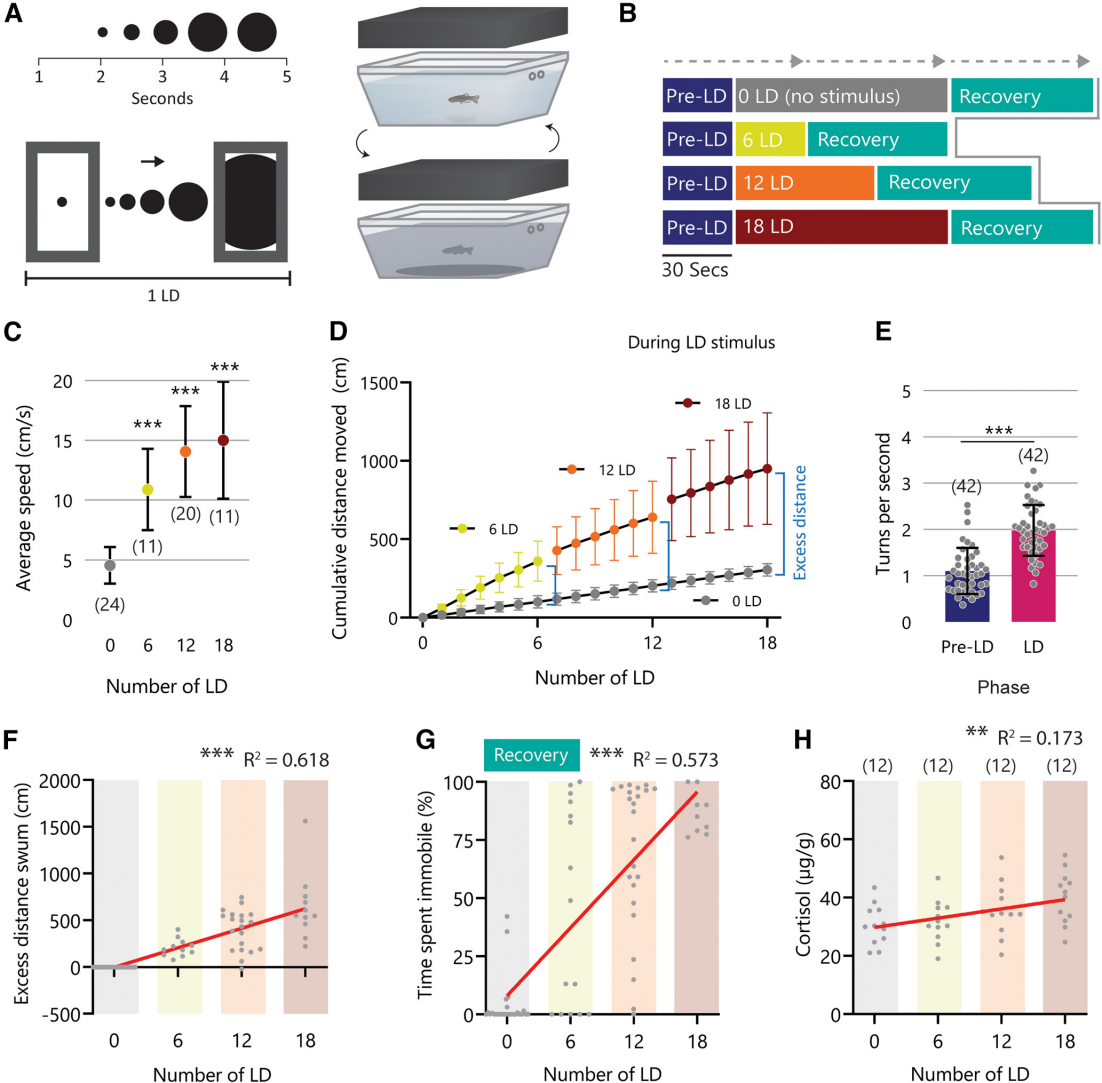
The LD stimulus presentation allows delivery of graded acute stress exposure. ***A***, The LD stimulus is presented on a monitor above the fish and each LD event lasts 4.8 s. ***B***, The LD is presented after a 30-s pre-LD phase and is then repeated 6, 12, or 18 times. A 30-s recovery phase is subsequently recorded. ***C***, The average speed during the LD phase for each intensity (cm/s). ***D***, Cumulative distance swum (cm) during the LD presentation for all LD groups. Excess distance (cm) is the δ between 0 LD and the cumulative distance at each LD number (6, 12, 18). ***E***, The number of turns (turns per second) during the first 6 LD in the 6LD, 12 LD, and 18 LD group compared with the pre-LD phase. ***F***, Correlation between excess distance swum (cm) and the number of LD presented. ***G***, Correlation between time spent immobile (%) for the 60-s post-LD exposure and the number of LD presented. ***H***, Correlation between whole-body cortisol (μg/g) measured 15 min after LD exposure and the number of LD presented. Extended Data Figures 3-1 and 3-2 are available to support this figure. Error bars denote the mean ± 1 standard deviation (SD); significant post hoc comparisons and slope significance, **P < 0.01; ***P < 0.001. Abbreviations: LD, looming dot.

10.1523/ENEURO.0491-22.2023.f1-3 Extended Data Figure 1-3 Male and female adult zebrafish display social interaction. Time in the interaction zone (%) over 120 s for both female and male test fish with five female social cue fish (ns *p *>* *0.05). Download Figure 1-3, TIF file.

### Approach and interaction are dependent on the perceived salience of the social cue

To test whether the approach and interaction exhibited by the fish in our test represent a true social behavior rather than a novelty-related response to moving objects, we changed the salience of the cue and measured both approach and maintenance of the interaction. First, we compared different strains as the social cue, using the same strain as the test fish (TU), and two strains with different visual features. The Casper strain has a transparent appearance and the TL strain show larger fins. We then assessed the approach behavior by measuring both white zone preference and latency to approach social cues and normalized both measures to those of the TU fish (Fig. 2*A*). We found a significant difference in the approach behavior among the three strains (Kruskall–Wallis test, *H*_(3)_ = 10.18, *p *=* *0.003) with a significant difference between TU and TL (**p =* *0.004). Next, we assessed the maintenance of interaction by measuring both time in the interaction zone and the number of interactions of the three different strains (Fig. 2*B*). A significant difference among the strains was found (one-way ANOVA, *F*_(2,18)_ = 23.13; *p *<* *0.001) with clear differences between TU and TL (Tukey’s *post hoc* analysis; ***p <* *0.001) and TL and Casper (Tukey’s *post hoc* analysis; ***p <* *0.001). Next, we measured the effect of decreasing the number of conspecifics in the social cue tank. Approach was affected by the number of fish in the social cue tank (Kruskall–Wallis, *H*_(3)_ = 27.03, *p *<* *0.001). Fish spent more time with five fish compared with one fish (*p < 0.075) or when no fish (***p <* *0.001) was present (Fig. 2*C*). Maintenance of interaction was also affected by the number of conspecifics (one-way ANOVA, *F*_(2,38)_ = 128.9; *p *<* *0.001) with a reduction in maintenance with both no (***p <* *0.001) or one (***p <* *0.001) conspecific compared with five (Fig. 2*D*). Lastly, to determine whether approach and maintenance behavior can truly represent social behavior that requires reciprocal interaction, we assessed whether the conspecific fish used as a social cue exhibited signs of reciprocal behavior in the presence of the test fish. To this end, we measured the lateral distance (red double-headed arrow, Fig. 2*D*) between the test fish and the nearest social cue fish found within 1 cm of the wall separating them to determine whether the social cue fish also changes their relative position to be near to the test fish. The lateral distance between the test fish and the social cue fish on average is closer when the test fish is performing interactions compared with when it is not and in the white zone (Fig. 2*E*). The lateral distance was compared using 258 random interaction zone distances and 243 random noninteraction zone distances (Mann–Whitney test, *U*_(501)_ = 25 529, *p* < 0.001). The distances were normalized and plotted as a histogram where the test fish is 0 (Fig. 2*E*,*F*). Taken together these results argue that our behavioral paradigm measures true social behavior involving both a drive to approach social cues and the ability to maintain reciprocated social interaction.

### A new delivery method for inducing graded acute stress exposure in adult zebrafish

To measure the effects that acute stress has on social behavior, we designed an acute-stress paradigm that mimics an aerial-looming-predator to induce stress in adult zebrafish. This paradigm consists of a small tank placed underneath a Raspberry Pi display controlled via a custom-built graphical user interface (GUI) on a master PC to generate and present graphics interchange format (GIF) images (Fig. 3*A*). The looming dot (LD) stimulus is an expanding black circle on a white background that starts at the center and fills most of the display. The custom-built GUI control panel generates a short sequence, which contains a list of 15 parameters that can be varied. We tested several parameters including the looming speed and greyscale intensity of the LD (Extended Data Fig. 3-1) but found changing the number of sequential LD to be the most effective means to control the intensity of the stimulus and evoke a graded response. We refer to this as LD number, which changes both the number of stimuli and the total duration of the stressor exposure (Fig. 3*B*). First, to measure meaningful behavioral changes associated with LD, subjects were recorded for 30 s to determine baseline (pre-LD) measurements, and then while being exposed to one of four LD intensities: 0 LD (handling control), 6 LD, 12 LD, or 18 LD (Fig. 3*B*). During LD presentation, subjects displayed escape-like behavior characterized by an increase in speed. A one-way ANOVA followed by Dunnett’s multiple comparisons test revealed a significant increase in average speed during the presentation of the first 6 dots in all LD groups compared with the 0 LD group (*F*_(3,62)_ = 27.45; ***p <* *0.001; Fig. 3*C*). Although there was no difference in average speed among 6 LD-exposed, 12 LD-exposed, and 18 LD-exposed groups, the cumulative distance swum during the LD presentation increased relative to the number of LD as a result of an increase in the duration of the exposure. A repeated-measures one-way ANOVA of the 18 LD group following 6 LD (mean = 442.7 cm), 12 LD (mean = 712.6 cm), and 18 LD (mean = 949.7 cm) revealed a significant increase in distance moved (*F*_(1.029,10.29)_ = 43.19; ***p <* *0.001; Fig. 3*D*). Additionally, the average turn frequency increased during LD exposure from 1.09 to 1.97 turns per second during the first 6 LD compared with the pre-LD phase (two-tailed paired *t* test (*t*_(14)_ = 8.28, *p *<* *0.001; Fig. 3*E*). We performed linear regression and confirmed a significant correlation between the LD number and the excess distance swum (*R*^2^ = 0.618, *F*_(1,64)_ = 103.5, *p *< 0.001; Fig. 3*F*). During the LD exposure, fish performed an escape-like response. However, subsequently, subjects displayed intensity-dependent immobility after 6 LD, 12 LD, and 18 LD treatments. We performed linear regression and confirmed a significant correlation between the LD number and the time spent being immobile (*R*^2^ = 0.573, *F*_(1,73)_ = 97.94, *p *<* *0.001; Fig. 3*G*). To test whether stress-induced immobility was due to physical exhaustion or anxiety-related freezing, we treated fish with the anxiolytic compound Diazepam for 2 h before exposing them to 12 LD (Extended Data Fig. 3-2). Diazepam pretreatment inhibited immobility and treated fish spent less time immobile compared with the control group (*t* test; *t*_(14)_ = 7.9, *p *<* *0.001). Next, to assess whether LD exposure induced an endocrine stress response, we performed a cortisol assay to measure whole-body cortisol levels of the fish 15 min after being exposed to 0 LD, 6 LD, 12 LD, or 18 LD treatment. We found a significant correlation between the LD number and whole-body cortisol level (*R*^2^ = 0.173, *F*_(1,46)_ = 9.641, *P* =< 0.003; Fig. 3*H*). Collectively these results indicate that by varying the number of LD presented an increasingly higher level of acute stress exposure can be induced as evidenced by an increase in both endocrine and behavioral stress reactions.

10.1523/ENEURO.0491-22.2023.f3-1 Extended Data Figure 3-1 Changing grayscale intensity evokes an all-or-none response. ***A***, Speed increase presented as a fold increase compared to prelooming dot exposure. ***B***, Turn frequency presented as a fold-increase compared to the prelooming dot phase. ***C***, Time spent immobile during the 1-min poststress recovery phase. Download Figure 3-1, TIF file.

10.1523/ENEURO.0491-22.2023.f3-2 Extended Data Figure 3-2 Stress-induced immobility is inhibited by pretreatment with diazepam. Time spent immobile (%) after exposure to the looming dot acute stress stimulus (12 LD) with (Diazepam) and without (Ctrl) preexposure to Diazepam (156 nm) for 2 h (***p <* *0.001). Download Figure 3-2, TIF file.

### Acute stress modulates social approach and social maintenance in an intensity-dependent manner

**Figure 4.**
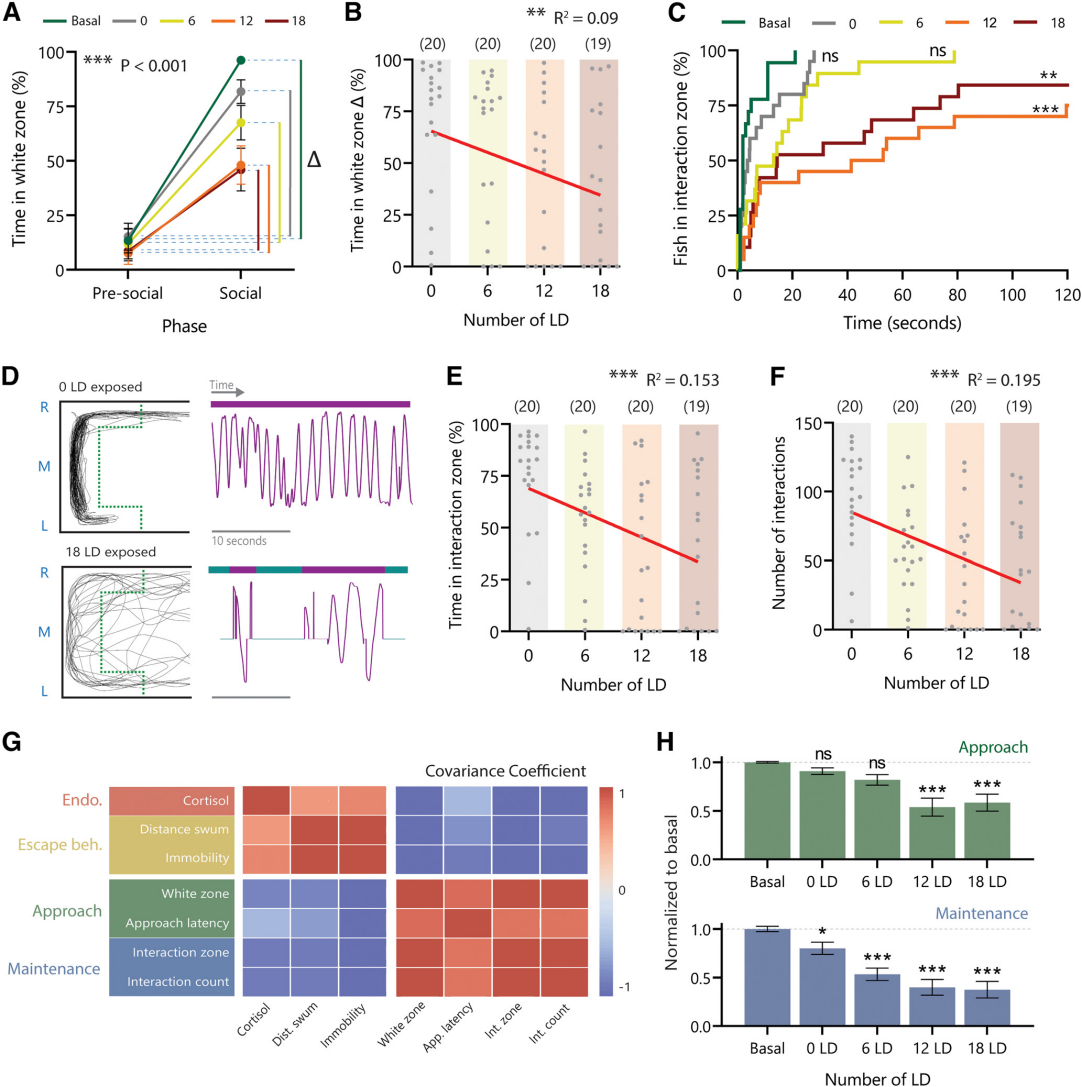
Social maintenance is modulated at a lower acute stress intensity than social approach. ***A***, Time spent in the white zone (%) with the visual divider present (presocial) and while the social cue is visible (social) and the difference (δ) for each LD group. ***B***, Correlation between the time in the white zone δ (%) and the number of LD presented. ***C***, The number of fish (%) arrived in the interaction zone for each LD group following the removal of the divider. ***D***, Example traces from above of a 0 LD and an 18 LD fish during the social phase and a 25-s example of the fish’ movement in the interaction zone on the L-R axis over time demonstrating the frequency of social interactions. ***E***, Correlation between the time spent in the interaction zone (%) and the number of LD presented. ***F***, Correlation between the number of interactions performed and number of LD fish were exposed to. ***G***, Covariance coefficient matrix for each behavior categorized into endocrinological measures, escape behaviors, social approach and social maintenance. Covariance was measured across all treatment groups. ***H***, Social approach and social maintenance measures normalized to basal for each LD intensity. Extended Data Figures 4-1, 4–2, and 4-3 are available to support this figure. Error bars denote the mean ± 1 standard deviation (SD); significant post hoc comparisons and slope significance, ns *P* > 0.05, *P < 0.05 ,**P < 0.01; ***P < 0.001. Abbreviations: LD, looming dot.

To measure the effects of acute stress intensity on approaching social cues, we measured the time spent in the white zone and the latency to approach the social cue on exposure to different numbers of LD. The time in the white zone increased significantly when the social cue was present but the magnitude of the difference between the time spent in the white zone before and after the social cue presentation was dependent on the LD number (Fig. 4*A*). A two-way ANOVA revealed a significant phase effect (*F*_(1,186)_ = 186, *p *=* *0.001) and a significant interaction between phase and LD number (*F*_(1,186)_ = 4.06, *p *= 0.004). There was a significant correlation between the number of LD fish were exposed to and the difference in time spent in the white zone (*R*^2^ = 0.09, *F*_(1,77)_ = 7.696, *p *=* *0.007; Fig. 4*B*). The LD number also had a significant effect on the latency to enter the interaction zone once the social phase was initiated (Kruskall–Wallis test, *H*_(5)_ = 22.26, *p *<* *0.001), and there was an increase in latency in the 12 LD group (*p *<* *0.001) and the 18 LD (*p *=* *0,002) group compared with the basal group (Fig. 4*C*). The ability to approach social cues requires the ability to both recognize the social cue as well as a drive to be near the social cue. To distinguish between these two possibilities, we tested whether LD exposure impacted fish’s ability to recognize its conspecific. Even after the highest intensity 18 LD exposure, the fish changed their head direction when presented with the social cue indicating that the stress exposure did not compromise their ability to recognize the social cue (Extended Data Fig. 4-1).

10.1523/ENEURO.0491-22.2023.f4-1 Extended Data Figure 4-1 After being exposed to high intensity stress, fish still orientate towards the social cue. ***A***, Heading direction of the test fish measured as >90° or <90°. ***B***, Time spent <90° (facing social cue) for 18 LD exposed fish in the presocial phase and social phase. ***C***, Number of fish in the <90° direction following 18 LD exposure in the last 10 s of the presocial phase and the social phase (*p <* *0.05). Download Figure 4-1, TIF file.

10.1523/ENEURO.0491-22.2023.f4-2 Extended Data Figure 4-2 LD-exposed groups exhibit reductions in social approach and maintenance compared to untreated fish. ***A***, Time in the interaction zone (%) over 120 s for each treatment group. ***B***, The number of interactions performed in the interaction zone during the 120-s social phase (ns *p *>* *0.05; ***p <* *0.001). LD exposure had a clear effect on the time spent in the interaction zone (Kruskall–Wallis, *H*_(5)_ = 42.38, *p* < 0.001) with 6 LD, 12 LD and 18 LD showing reduction compared to the basal group (Dunn’s multiple comparisons (*p* < 0.001) for each of the three comparisons. The number of interactions performed also was affected by LD exposure (one-way ANOVA, *F*_(4,92)_ = 8.2, *p *<* *0.001) with 6 LD, 12 LD, and 18 LD groups all performing fewer interactions compared to the Basal group [Dunnett’s multiple comparisons test (*p* < 0.001) for each of the three comparisons]. Download Figure 4-2, TIF file.

10.1523/ENEURO.0491-22.2023.f4-3 Extended Data Figure 4-3 Distance swum reduces in the social approach and maintenance following stress. Distance swum in meters in the social approach and maintenance assay following different intensities of stress exposure (ns *p *>* *0.05; **p <* *0.01, ***p <* *0.001). Download Figure 4-3, TIF file.

Next, we measured how fish performed and maintained social interactions for the duration of the trial after being subjected to LD exposure. Example traces of a fish exposed to 0 LD or 18 LD indicated that the stereotypical “C” shape pattern as well as the frequency of interactions are compromised in the 18 LD-exposed group (Fig. 4*D*). There was a significant difference in the time spent in interaction zone (%) between the basal and LD-exposed group (Extended Data Fig. 4-2*A*) and a significant correlation between LD number and the time spent in the interaction zone (*R*^2^ = 0.153, *F*_(1,77)_ = 13.90, *p *<* *0.001; Fig. 4*E*). Further, there was a significant difference in the number of interactions between the basal and LD-exposed group (Extended Data Fig. 4-2*B*) and a significant correlation between the number of LD and the number of interactions performed (*R*^2^ = 0.195, *F*_(1,77)_ = 18.59, *p *<* *0.001; Fig. 4*F*). A reduction in social approach and interaction following LD exposure was not because of LD-induced freezing. Fish were still mobile following LD exposure although a reduction in distance swum during the trial was measured in the 6 LD (*p *=* *0.001), 12 LD (*p *<* *0.001), and 18 LD (*p *< 0.001) groups (one-way ANOVA, *F*_(4,93)_ = 11.93, *p *<* *0.001; Extended Data Fig. 4-3).

Overall, as the LD number increased the endocrine and behavioral stress measures increased while the social behavior measures decreased. Endocrine and stress behavior measures had a negative covariance with both social approach and social maintenance measures across all of the treatment groups (Fig. 4*G*). Interestingly, the extent of this negative covariance was different between the social approach and social maintenance at each treatment level. Social approach was affected by LD exposure (one-way ANOVA, *F*_(4,92)_ = 9.89, *p *<* *0.001), but it was only affected at higher intensities represented by 12 LD and 18 LD [Šídák’s multiple comparisons *post hoc* analysis for 12 LD (*p *<* *0.001) and 18 LD (*p *<* *0.001)]. In contrast, while social maintenance was also affected by stress (one-way ANOVA, *F*_(4,92)_ = 9.89, *p *<* *0.001) significant changes in behavior were observed at every treatment level [Šídák’s multiple comparisons *post hoc* analysis, 0 LD (*p *<* *0.05), 6 LD (*p *<* *0.001), 12 LD (*p *<* *0.001) and 18 LD (*p *< 0.001)]. This result indicates that social maintenance is affected at a lower level of acute stress exposure than social approach.

## Discussion

Here, we combined a novel behavior test and a new acute stress delivery method to demonstrate that acute stress modulates social approach and maintenance in an intensity-dependent manner in adult zebrafish. Strikingly our results suggest that distinct steps in social behavior are differentially susceptible to the effects of acute stress exposure.

The link between stress exposure and social behavior has been documented in many species including humans. However, social behavior was measured at different time points, using different types of stressors in different contexts, which produced a complex range of outcomes. In humans, acute stress initially increases motivation to perform pro-social and social approach behavior (von Dawans et al., 2012; Buchanan and Preston, 2014). However, this effect diminishes over time (Margittai et al., 2015) and even reverses (Vinkers et al., 2013). The prosocial effect also depends on the type of stressor an individual is exposed to (von Dawans et al., 2018). Further, exposure to stress leads to a variety of outcomes depending on the societal context (Davidson et al., 1991; Wilkinson, 2004; Kendrick et al., 2012).

Similarly, rodents exhibit social behavior that is modulated by stress and many studies have investigated the effects that different stressors have on social behavior. Often studies assessed unfamiliar male-to-male dyadic interactions, which mainly results in an increase in aggressiveness or social avoidance rather than pro-affiliative behaviors (Toth and Neumann, 2013). This outcome has been observed by both acute threat (File and Hyde, 1978; de Almeida and Miczek, 2002) and by prolonged stress administered at different developmental time points including prenatal (de Souza et al., 2013), neonatal (Wei et al., 2013), peripubertal (Márquez et al., 2013), and adult (van der Kooij et al., 2014a, b). Activation of the hypothalamic-pituitary-adrenal (HPA) axis by corticosterone administration alone can also induce similar outcomes (Leshner and Schwartz, 1977; Mikics et al., 2004; Veenit et al., 2013). It is well established that motivation to engage in pro-social behavior is diminished in more hostile environments (File and Hyde, 1978). Therefore, when tested in a setting where the social cue is familiar and accessible, a group of freely moving familiar cage-mates exhibit pro-social behavior in the form of huddling in response to a potential threat (Kendig et al., 2011; Bowen et al., 2013).

Given the complexity of the effect of stress on social outcomes, it is critical to control relevant parameters involving both stress exposure and social behavior. A crucial variable in assessing social behavior is the use of specific behavioral assays. Social functioning involves distinct behavioral sequences and a large number of social behavior assays available in rodents test different aspects of social behavior. A widely used social interaction test allows interaction of a test subject and a social cue without a physical divider or spatial restriction (File and Hyde, 1978). In contrast, the 3-chambered social approach test (Nadler et al., 2004) and the social avoidance-preference test (Berton et al., 2006) measure approach to a conspecific in a neutral environment and subsequent interaction with a conspecific in a restricted space. Social approach in these assays is usually measured as time spent in proximity to the social cue (Brodkin et al., 2004). The major difference between the way social behavior is measured in these studies compared with ours is that by including the white zone as a barrier, which needs to be overcome to initiate approaching the conspecific, we can distinguish the approach versus the interactions and the maintenance of those interactions. In our tank, there was no intrinsic area bias for the left or right side of the tank (Extended Data Fig. 1-2). However, white was the least desirable color when compared with blue, red, or yellow. Several previous studies have investigated color preference in zebrafish and found different preference depending on specific contexts, experimental paradigms and conditions (Avdesh et al., 2012; Bault et al., 2015; J. Oliveira et al., 2015; Park et al., 2016). For example, red has been found to be aversive (van Staden et al., 2020) or preferred (Siregar et al., 2020). These results indicate that strong context-dependent color preference exist in zebrafish. In our experimental set up, white was the least prefered when compared with other colors. In our assay, we combined two independent measures to obtain the social approach score and the social maintenance score. Social approach requires the fish to not only initially approach the social cue but also to maintain a change in the zone preference. Social maintenance requires the fish to maintain close proximity and perform active social behavior in the form of interactions. Social maintenance in our assay is a reflection of sustained active social behavior that goes beyond mere spatial preference, comparable to nose contacts in rodent assays (Mines et al., 2010).

Social deficits in stressed fish have been observed but differing outcomes have been reported. For example, if fish were acutely stressed and placed in an open tank with free-swimming conspecifics, an increase in shoal density is observed (Miller and Gerlai, 2007; Green et al., 2012; Kleinhappel et al., 2019). However, in a tank in which the stressed subject must approach a physically separated visual social cue, a reduction in socializing is observed (Abreu et al., 2016b; Giacomini et al., 2016; Barcellos et al., 2020). Similarly, in a three-chamber social interaction assay under aversive lighting conditions, zebrafish displayed no preference for the social cue, but an increase in social preference was observed following administration of the anxiolytic compound Buspirone (Barba-Escobedo and Gould, 2012). Chronic stress studies in zebrafish have led to even more divergent outcomes: during a chronic unpredictable stress protocol, subjects displayed a loosening of group cohesion after each stressor (Pavlidis et al., 2015). The same outcome was observed after 14 d of unpredictable chronic stress, but not after 7 d (Piato et al., 2011). A different type of chronic stress, early social deprivation, led to a reduction in social preference (Tunbak et al., 2020). However, in these previous investigations into the effects of stress on social behavior in zebrafish, it has been difficult to control two important parameters namely stressor intensity and specific steps in social behavior that are demonstrated and measured.

To measure a drive to approach a social cue, we created a conflict between unfavorable and attractive stimuli, where a subject is required to invest resources or effort to overcome an unfavorable cue to interact with the reward. In this test, a conflict between the avoidance of the white zone and the innate drive to approach a social cue attest to the strength of the individual’s social preference. First, we demonstrate that the perceived salience of the social cue moderates the drive to approach and maintain proximity to the social cue. Social preference is affected by both the strain of the conspecifics and the number of conspecifics. In the less preferred scenario comprising of one fish or the TL strain, the innate avoidance of the white zone outweighs the drive to interact with the social cue. Next, we demonstrate how fish exposed to LD acute stress alter their social approach and maintenance behaviors. At lower intensities of stress, subjects displayed a mild reduction in approaching the social cue and entering the white zone, while at higher intensities, they largely kept away from the white zone. A consistent result was obtained in a previous study, which used the color red as an aversive cue paired with a social cue. Here, subjects initially did not approach the cue, but this trend could be reversed using dopaminergic and serotonergic manipulation (van Staden et al., 2020). A conflict could also be generated between two rewards, in which zebrafish show a less vigorous, but a more persistent tendency to approach a social reward compared with food (Daggett et al., 2019). Studies show, that similar to rodents, approaching a social cue is largely governed by motivation-associated mechanisms in zebrafish (Kareklas et al., 2023). When zebrafish approached a social cue, a significant increase in dopamine and DOPAC was measured, indicating an increase in dopaminergic activity (Saif et al., 2013). Polymorphisms in dopamine precursor and dopamine receptor genes are associated with exploration-sociability motivational phenotypes in zebrafish (Gonçalves et al., 2022). Furthermore, the ablation of dopaminergic neurons leads to social deficits similar to those observed in stressed fish (Saszik and Smith, 2018). The association between dopaminergic activity and social behavior supports that approaching a social cue is a motivation-driven, goal-directed behavior.

In conclusion, our results demonstrate that acute stress reduces both social approach and social maintenance in adult zebrafish. Interestingly, lower stress intensity reduces social maintenance without affecting the social approach, while a higher stress level is required to alter social approach. These findings provide evidence that acute stress differentially modulates distinct steps in social functioning in an intensity-dependent manner in adult zebrafish. Determining the effects of different levels of acute stress exposure on distinct steps of social behavior is an important prerequisite in identifying correct neural correlates of how stress modifies social behavior.

## Synthesis

Reviewing Editor: David Schoppik, New York University Decisions are customarily a result of the Reviewing Editor and the peer reviewers coming together and discussing their recommendations until a consensus is reached. When revisions are invited, a fact-based synthesis statement explaining their decision and outlining what is needed to prepare a revision will be listed below. The following reviewer(s) agreed to reveal their identity: NONE.

This manuscript titled “Acute stress modulates social approach and social maintenance in adult zebrafish” establishes a new assay for assessing social behavior and a new approach for varying the degree of stressors, and uses these two novel principles to examine the relationship of different degrees of stress on social approach and maintenance. The effect of stress on social behavior is well documented and is an important social and biomedical consideration. While the relationship between the two is well documented, less is clear about how the degree of stress manifests itself on social behavior. Zebrafish are a powerful model for addressing this concern, and the assays established here are useful in the field. The main finding of the study is that stress intensity has more robust effects on approach than maintenance, though the contribution of the assays outlined in the study are also a considerable and noteworthy point. While we are excited by the study, and we find the results interesting, we have some concerns with the establishment of the two assays. These concerns not only relate to the findings of the study, but to the longer-term usage of the assays described. Many of the concerns can likely be addressed by a more elaborate explanation in the writing.

1. The authors state that (line 89) “fish have to overcome an aversive area.” We presume this is reference the white, but white is not in its own right aversive. Just because fish like blue more than white doesn‘t mean white is “aversive.” This is not just a picky point, but it is central to their premise that fish overcome an aversive barrier to see conspecifics.

2. In lines 105-108: It is unclear what these two sentences mean. “The average time that test fish take from first leaving the blue zone to entering the interaction zone is 2.21 seconds, indicating that fish leave the blue zone in order to approach the social cue. With the divider removed, 94.7% of fish leave the blue zone within 12 seconds to enter the white zone (Figure 1D).” In appears to say first that fish leave the blue zone in 2 seconds, and the following sentence says 12. We assume these two are different measures, but it is not clear how they differ or what each value represents.

3. For figure 1, (lines 93-95) it isn’t clear how the control was performed. It seems like fish were allowed to swim freely for 10 min with a divider in place. While there is value in this measurement as it informs as to the baseline behavior, it does not account for the experimenter removing the divider or any other parameter in the actual experiment. We feel that the correct control should be a 3 min experiment where the divider removed after 30 sec and fish are exposed to another tank with no fish (i.e., treated exactly the same, except no conspecifics are present in the adjacent tank).

4. Along the same lines, we’d like to see that ‘blue’ in this context is truly attractive and it the effect isn’t just being positioned away from a barrier or some other confound. What would happen if the area was black (which should also be attractive), red (which presumably would be more difficult to see), or green (which I assume is neutral). It may also be interesting to see what happens in a similar set up, except with no color difference. We know the authors will not want to do all of these, but just some determination that blue is attractive and that fish are choosing a conspecific over an attractive color. These controls are important when establishing a novel assay, and are central to the conclusions of the authors.

5. For figure two, it is unclear why these data are all ‘normalized’ and what normalized actually means. Can’t the authors just measure exactly as they did in fig 1 and then do statistics on those raw numbers?

6. For figs 2a-b, what is actually being measured as a reflection of ‘approach’ and ‘maintenance’? We assume ‘maintenance’ is the number of L-R interactions, as in fig 1f, but approach is less clear to us.

7. In lines 162-164, the authors state that they tested several LD parameters and found number of sequential LDs to be ‘particularly’ effective. What does this mean? How was efficacy measured? What parameters were tested, and what were the results from these? Again, as this is a novel application of LD and the central premise is that the intensity (as assessed as number of sequential LDs presented) is related to the degree of stress-induction, a clear description of what is being measured is essential to a reader assessing (and replicating) the work.

8. We are concerned about the regressions of locomotor behavior (e.g. 4B, 4E, 4F) where there are fish that are largely if not entirely immobile. Those fish would just add a “0” to any metric and drag down the average, but those fish aren’t competent to respond to the stimulus. I’m not sure how many of the results would obtain if regressions were restricted to fish that were actually motile.

Minor points:

1. There is a certain vagueness when the authors are talking about aggression, esp. in the introduction, and we encourage them to be a bit clearer. For example, the first paragraph (lines 32-36) discuses findings where acute stress “reduced social interaction while increasing aggression”, yet aggression is a social behavior that requires interaction. The authors state this directly in lines 46-47, and thus we are not sure what this sentence means aside from an abstract “some social cues increase and others decrease”. It’s a very small point, but we found ourselves trying to determine what they meant by aggression throughout.

## Author Response

1. The authors state that (line 89) “fish have to overcome an aversive area.” We presume this is reference the white, but white is not in its own right aversive. Just because fish like blue more than white doesn’t mean white is “aversive.” This is not just a picky point, but it is central to their premise that fish overcome an aversive barrier to see conspecifics. We appreciate the reviewer’s comment and agree that white zone cannot be considered inherently “aversive.” To address both point 1 and point 4, we carried out further experiments testing fish’s color preference in the context of our test tank. Firstly, we demonstrate that there are no inherent biases within the arena, and fish spend an equal amount of time in the left and right sides of the tank with a general preference for the middle (Zone 2 and 3) when there are no different colored zones (new Extended data figure 1‐1). Subsequently, we demonstrate that fish favour any colour (blue, green, red, yellow) over white (new Extended data figure 1‐2). Our new results demonstrate that white is indeed least favorable compared to blue or other colors in our test tank. We therefore changed the text and replaced the word “aversive” with “unfavorable.” To measure an animal’s drive or motivation, a cost to gain access to reward is typically measured. Using an “unfavorable” cue we can still measure a cost to gain access to social reward. It is not necessary that the cost is measured using an inherently “aversive” cue. In addition to the supplementary data, updates in the methods, results, and discussion have been added pertaining to this new result. Especially in the discussion (lines 299‐306), we describe the results of other published studies with color preference test and point out that color preference is context‐dependent and that in our particular tank design, white was the least preferable.

2. In lines 105‐108: It is unclear what these two sentences mean. “The average time that test fish take from first leaving the blue zone to entering the interaction zone is 2.21 seconds, indicating that fish leave the blue zone in order to approach the social cue. With the divider removed, 94.7% of fish leave the blue zone within 12 seconds to enter the white zone (Figure 1D).” In appears to say first that fish leave the blue zone in 2 seconds, and the following sentence says 12. We assume these two are different measures, but it is not clear how they differ or what each value represents. We agree that these sentences are confusing and thank the reviewer for pointing this out. It is correct that 2.21 and 12 seconds refer to different measures. We have modified the sentences in the result section to make this clearer (lines 113‐116). “The average time that test fish take from first leaving the blue zone, traversing the white zone and entering the interaction zone is 2.21 seconds, indicating that fish leave the blue zone in order to approach the social cue. For 94.7% of fish, this action was initiated within 12 seconds from the point when the divider has been removed (Figure 1D).”

3. For figure 1, (lines 93‐95) it isn’t clear how the control was performed. It seems like fish were allowed to swim freely for 10 min with a divider in place. While there is value in this measurement as it informs as to the baseline behavior, it does not account for the experimenter removing the divider or any other parameter in the actual experiment. We feel that the correct control should be a 3 min experiment where the divider removed after 2 30 sec and fish are exposed to another tank with no fish (i.e., treated exactly the same, except no conspecifics are present in the adjacent tank). Demonstrating the prolonged baseline behaviour was the intent of the data in Figure 1B. The controls that the reviewer have recommended are presented in Figure 2C and 2D.

4. Along the same lines, we’d like to see that ‘blue’ in this context is truly attractive and it the effect isn’t just being positioned away from a barrier or some other confound. What would happen if the area was black (which should also be attractive), red (which presumably would be more difficult to see), or green (which I assume is neutral). It may also be interesting to see what happens in a similar set up, except with no color difference. We know the authors will not want to do all of these, but just some determination that blue is attractive and that fish are choosing a conspecific over an attractive color. These controls are important when establishing a novel assay, and are central to the conclusions of the authors. We agree with the reviewer about the importance of this experiment. Therefore we have carried out new experiments to demonstrate that blue is attractive/favorable. This experiment is described in our response to point 1 and is included as a new supplementary figure 1 and 2.

5. For figure two, it is unclear why these data are all ‘normalized’ and what normalized actually means. Can’t the authors just measure exactly as they did in fig 1 and then do statistics on those raw numbers? Both social approach and maintenance scores in Figure 2 represent combinations of two independent measures as described in the result section. To quantify social approach we utilize average normalized score for the time spent in the white zone (%) and the latency to approach the interaction zone (s) while social maintenance is an average normalized score for the time spent in the interaction zone (%) and the number of interaction performed. We have added the following paragraph in the methods section to explain how normalization was done. “To normalize the data, first, the average readout for each measure was calculated for the ‘basal’ group, giving the ‘basal average’. Each individual point was then divided by the basal average to give a ‘normalized readout’, with 1 being equal to the basal average. Next, the average normalized readout for time in the white zone and latency to approach the interaction zone was calculated to give an approach score for each fish ((time in the white zone/ latency to approach the interaction zone)/2). The same calculation was performed for social maintenance using the time spent in the interaction zone and the number of interactions ((time spent in the interaction zone / number of interactions)/2).”

6. For figs 2a‐b, what is actually being measured as a reflection of ‘approach’ and ‘maintenance’? We assume ‘maintenance’ is the number of L‐R interactions, as in fig 1f, but approach is less clear to us. 3 As described above for the point 5, approach measures time spent in the white zone (%) plus the latency to approach the interaction zone (s) while social maintenance is an average normalized score for the time spent in the interaction zone (%) and the number of interaction performed. This is described in the result section.

7. In lines 162‐164, the authors state that they tested several LD parameters and found number of sequential LDs to be ‘particularly’ effective. What does this mean? How was efficacy measured? What parameters were tested, and what were the results from these? Again, as this is a novel application of LD and the central premise is that the intensity (as assessed as number of sequential LDs presented) is related to the degree of stress‐ induction, a clear description of what is being measured is essential to a reader assessing (and replicating) the work. We have included additional data addressing this point (new Extended data figure 3‐1). In this data we show that changing grayscale intensity, one of the aforementioned parameters, did evoke differences in response alut the response was binary rather than graded. At 25% grayscale fish did not respond to the stimulus whereas at 50%, 75% and 100% the response was of equal magnitude, demonstrating an ‘all or nothing’ response rather than the required graded response. Updates in the methods (lines 444‐447) and results (lines 170‐ 171) have been included to describe this data.

8. We are concerned about the regressions of locomotor behaviour (e.g. 4B, 4E, 4F) where there are fish that are largely if not entirely immobile. Those fish would just add a “0” to any metric and drag down the average, but those fish aren’t competent to respond to the stimulus. I’m not sure how many of the results would obtain if regressions were restricted to fish that were actually motile. This is an important point for clarification. In Figure 4B, 4E and 4F we have measured time spent in white zone, interaction zone and number of interactions. The fact that many fish show 0 in these figures do not necessarily mean that they are not motile and incapable of responding. In order to make this point clear, we include a new data as supplementary figure 8 measuring fish’s motility in 120 sec social zone following an exposure to increasing level of LD. This data clearly shows that whilst mobility was decreased as a function of increasing LD exposure, the majority of fish are motile. Only at the highest intensity of stress, we observed instances of a few fish spending considerable amounts of time immobile and therefore inactive. We have included the data (new Extended data figure 4‐3) and complementary text results (236‐240).

Minor points:

1. There is a certain vagueness when the authors are talking about aggression, esp. in the introduction, and we encourage them to be a bit clearer. For example, the first paragraph (lines 32‐36) discuses findings where acute stress “reduced social interaction while increasing aggression”, yet aggression is a social behaviour that requires interaction. The authors state this directly in lines 46‐47, and thus we are not sure what this sentence means aside from an abstract “some social cues increase and others decrease”. It’s a very small 4 point, but we found ourselves trying to determine what they meant by aggression throughout. This is a fair and important criticism that has been addressed by making a small adjustment to the text: “In rodents, studies suggest that acute stress exposure leads to reduced pro‐social behaviours in terms of social motivation and interaction while increasing aggression (Anacker et al., 2016; Spencer, 2017). However, other studies demonstrate an increase in affiliative behavior and reduced aggression upon acute stress exposure (Wood et al., 2003; Muroy et al., 2016).”

We agree that aggression is a form of social interaction and the point being made in the text was to demonstrate the inconsistent findings regarding changes in social behaviour following stress.
