# Supplementary figures and images for: Acute Stress Modulates Social Approach and Social Maintenance in Adult Zebrafish
Source: eNeuro. 2023 Sep 8;10(9):ENEURO.0491-22.2023. doi: 10.1523/ENEURO.0491-22.2023 (PMC10493981; doi:10.1523/ENEURO.0491-22.2023)

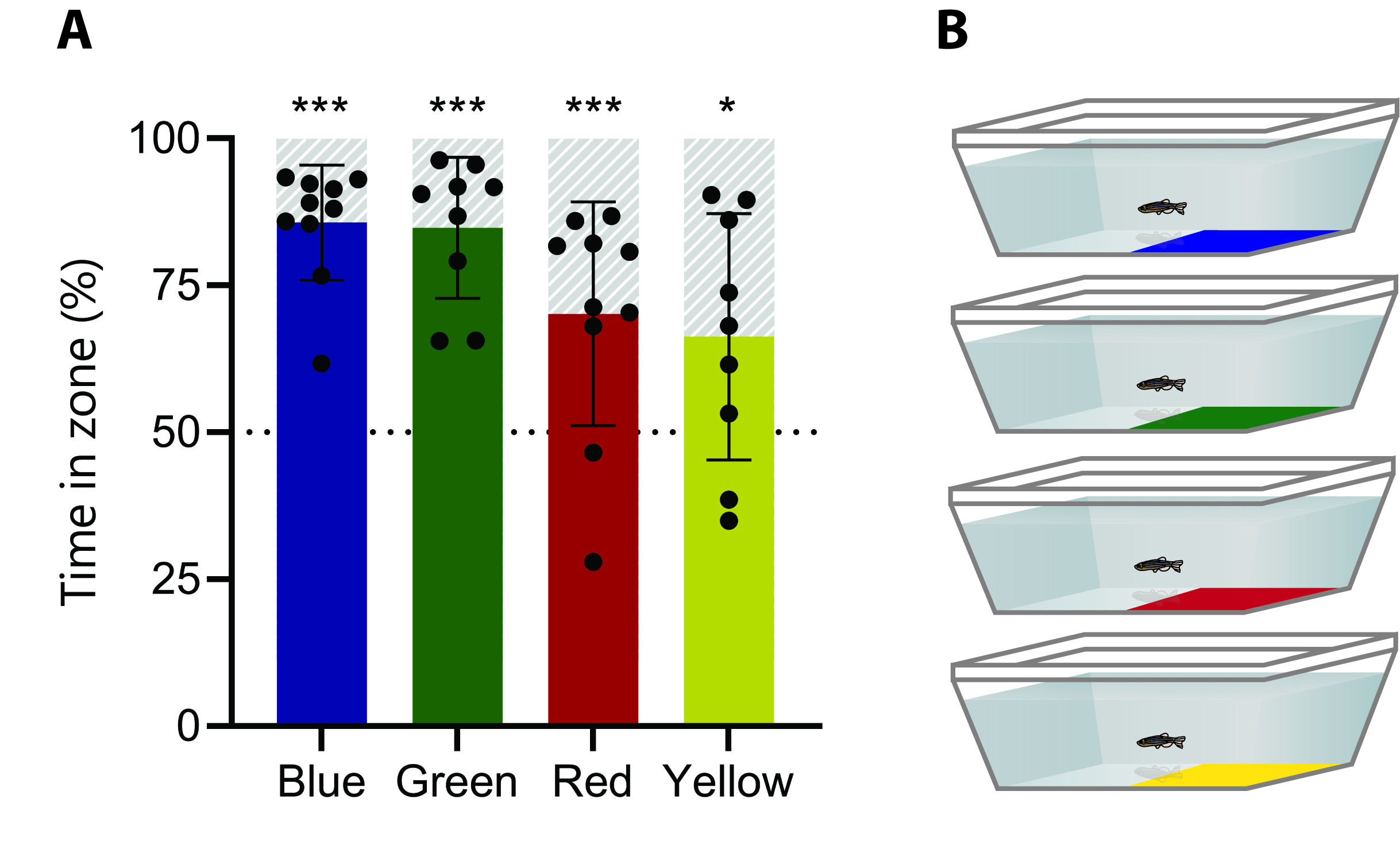

Supplement: Extended Data Figure 1-1 — White is least preferable compared to other colors. Time spent in the colored zone (blue, green, red, or yellow) compared to the white zone over a 5-min trial presented as a percentage of the trial duration (*p < 0.05, ***p < 0.001). Download Figure 1-1, TIF file. [file enu-eN-NWR-0491-22-s03.tif]

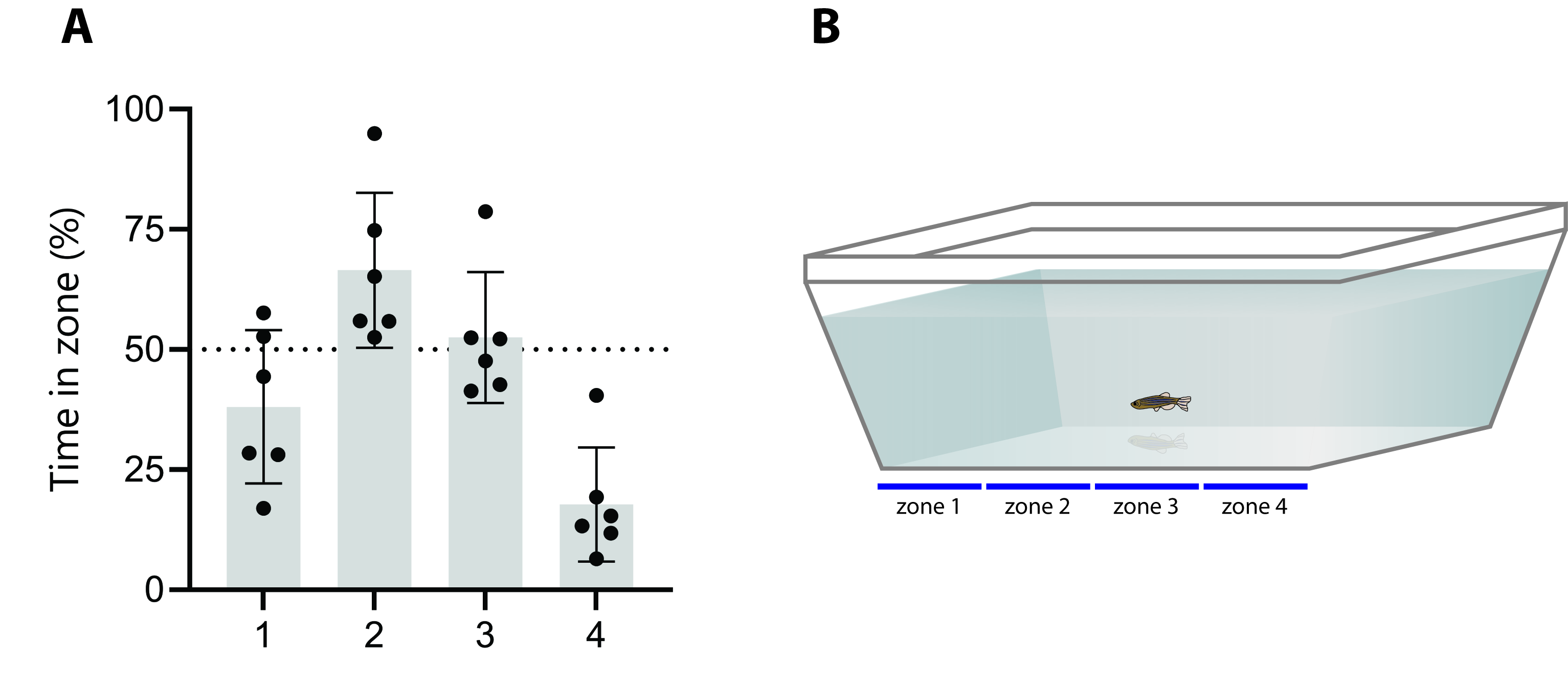

Supplement: Extended Data Figure 1-2 — There is no area bias in a neutral tank. Time spent in four equally divided zones 1, 2, 3, and 4 displayed as a percentage of the 5-min trial time. Download Figure 1-2, TIF file. [file enu-eN-NWR-0491-22-s04.tif]

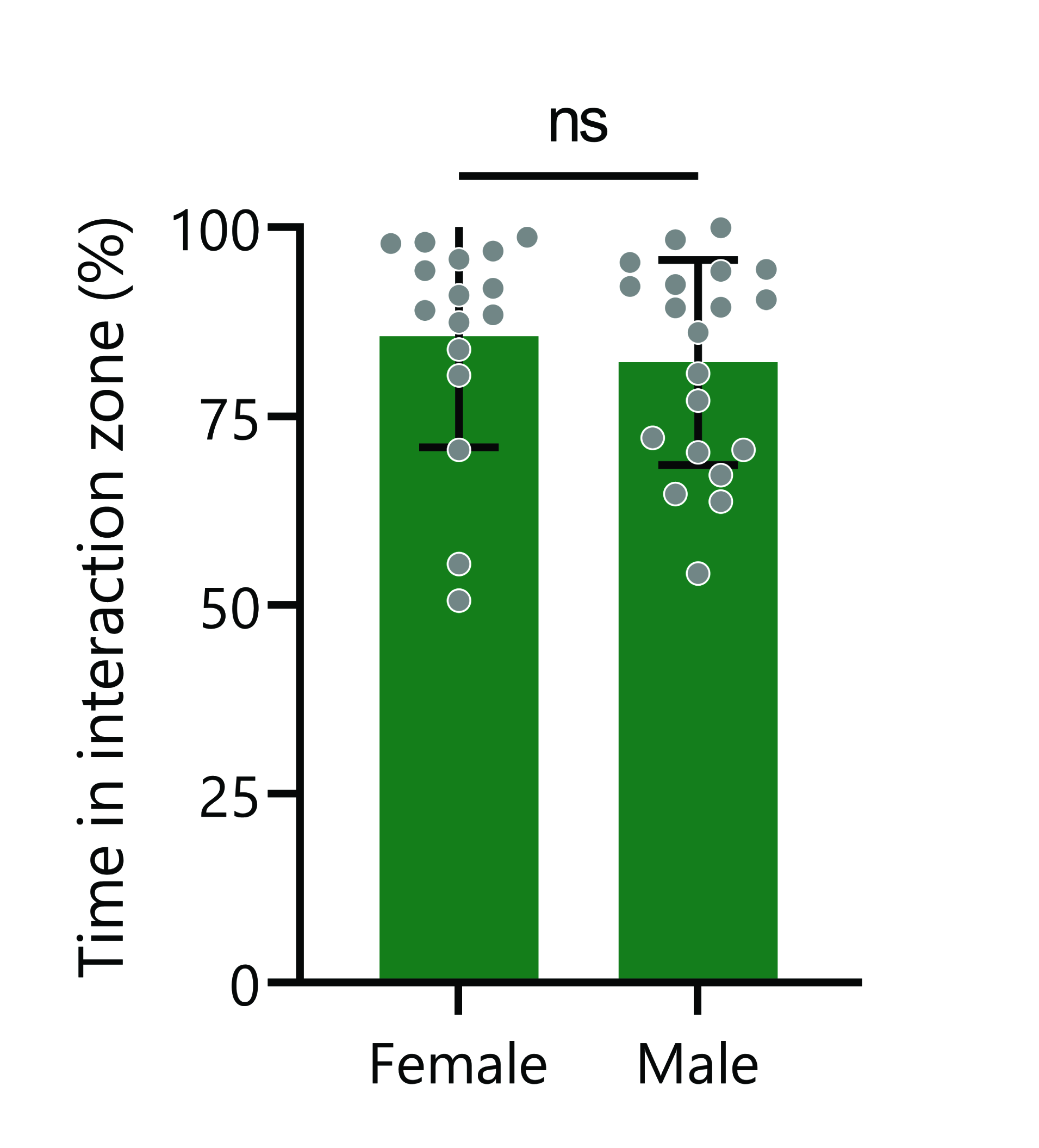

Supplement: Extended Data Figure 1-3 — Male and female adult zebrafish display social interaction. Time in the interaction zone (%) over 120 s for both female and male test fish with five female social cue fish (ns p > 0.05). Download Figure 1-3, TIF file. [file enu-eN-NWR-0491-22-s05.tif]

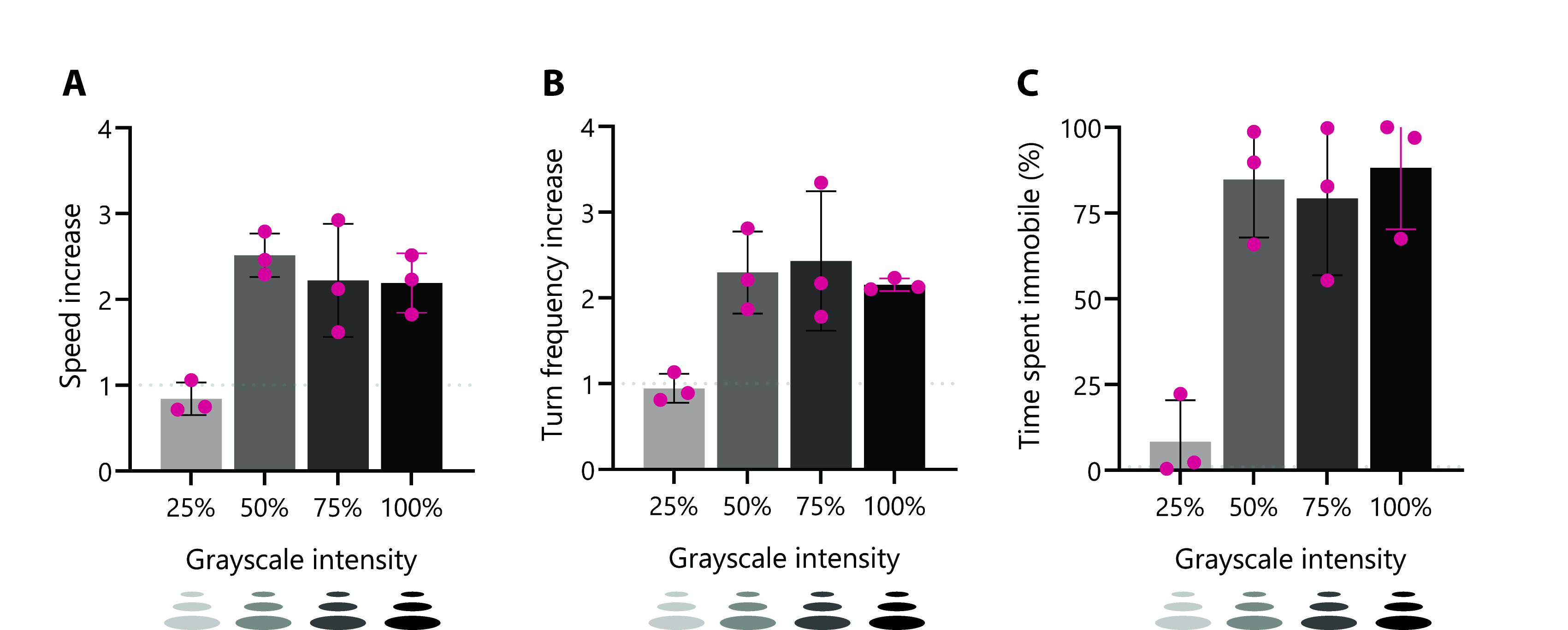

Supplement: Extended Data Figure 3-1 — Changing grayscale intensity evokes an all-or-none response. A, Speed increase presented as a fold increase compared to prelooming dot exposure. B, Turn frequency presented as a fold-increase compared to the prelooming dot phase. C, Time spent immobile during the 1-min poststress recovery phase. Download Figure 3-1, TIF file. [file enu-eN-NWR-0491-22-s06.tif]

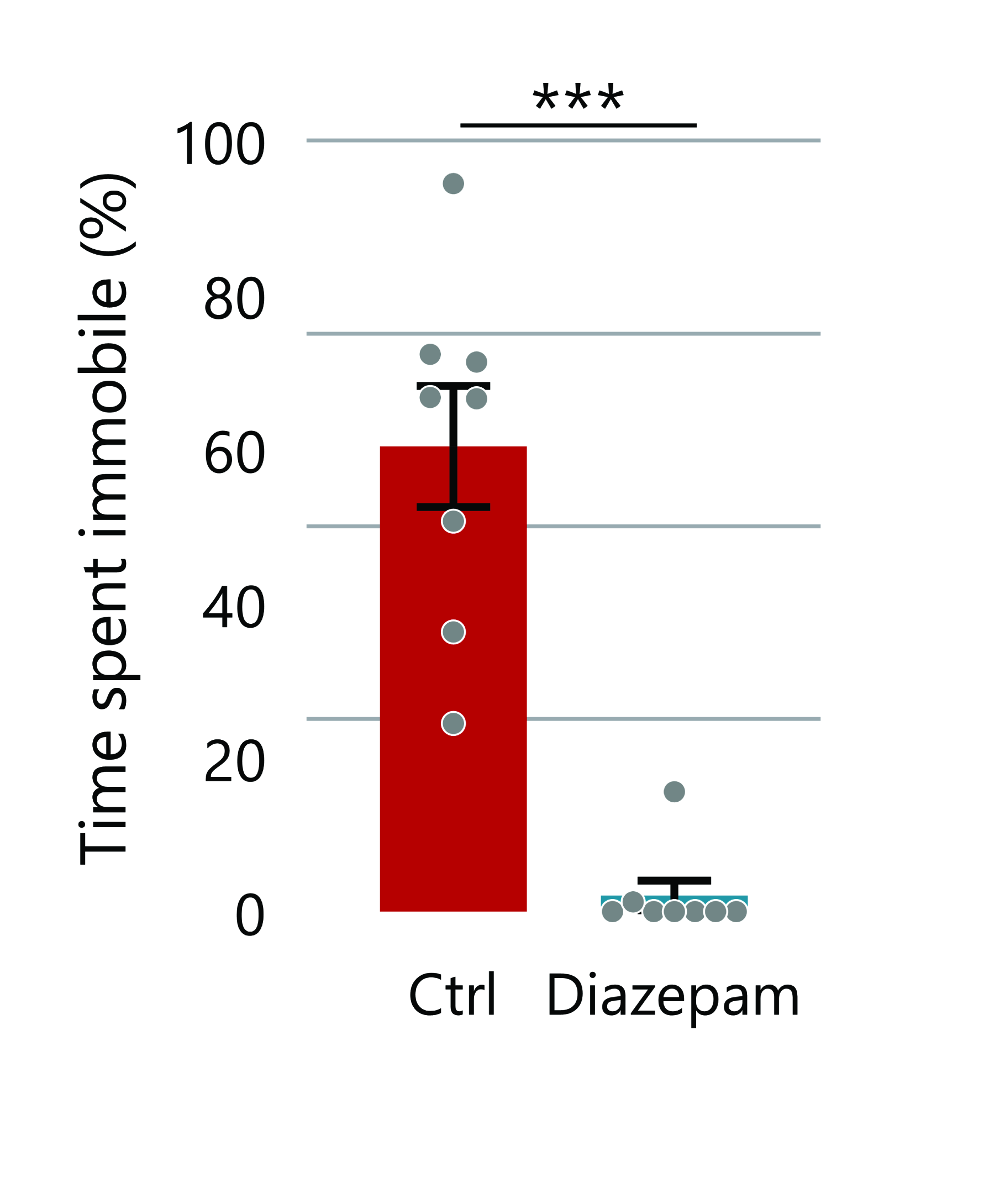

Supplement: Extended Data Figure 3-2 — Stress-induced immobility is inhibited by pretreatment with diazepam. Time spent immobile (%) after exposure to the looming dot acute stress stimulus (12 LD) with (Diazepam) and without (Ctrl) preexposure to Diazepam (156 nm) for 2 h (***p < 0.001). Download Figure 3-2, TIF file. [file enu-eN-NWR-0491-22-s07.tif]

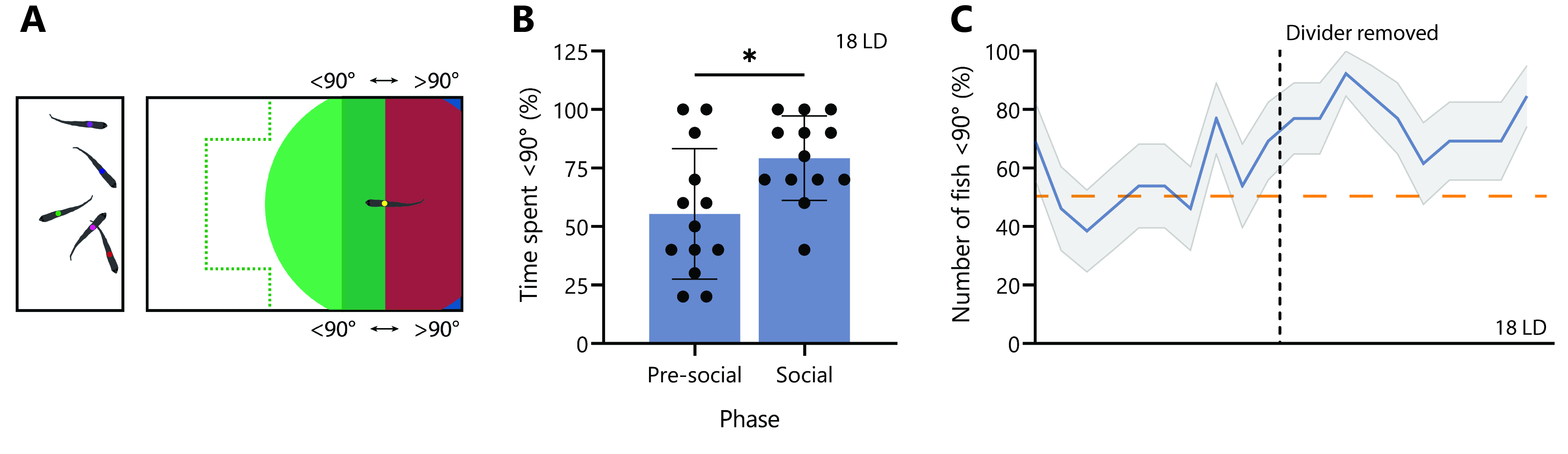

Supplement: Extended Data Figure 4-1 — After being exposed to high intensity stress, fish still orientate towards the social cue. A, Heading direction of the test fish measured as >90° or <90°. B, Time spent <90° (facing social cue) for 18 LD exposed fish in the presocial phase and social phase. C, Number of fish in the <90° direction following 18 LD exposure in the last 10 s of the presocial phase and the social phase (*p < 0.05). Download Figure 4-1, TIF file. [file enu-eN-NWR-0491-22-s08.tif]

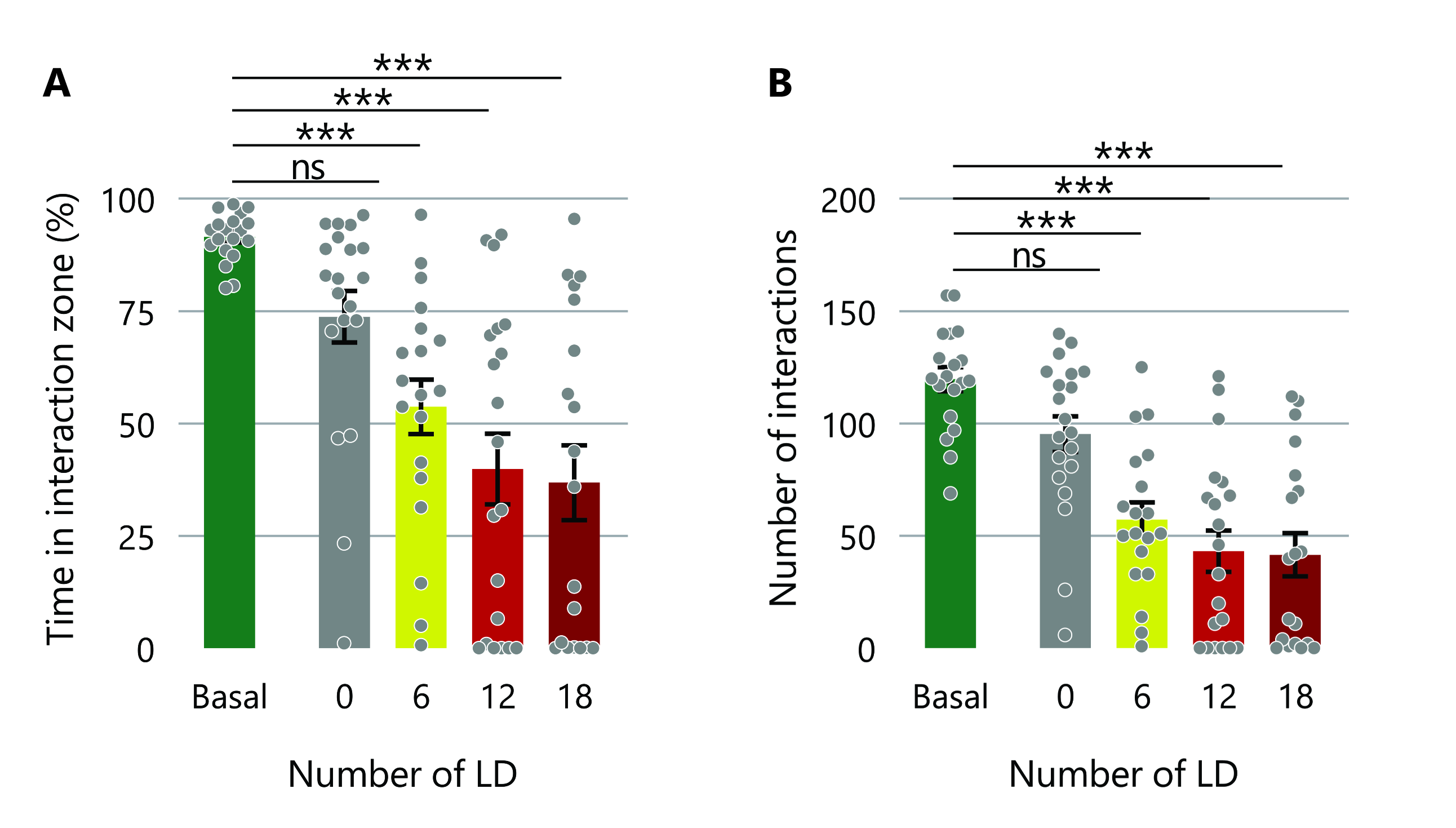

Supplement: Extended Data Figure 4-2 — LD-exposed groups exhibit reductions in social approach and maintenance compared to untreated fish. A, Time in the interaction zone (%) over 120 s for each treatment group. B, The number of interactions performed in the interaction zone during the 120-s social phase (ns p > 0.05; ***p < 0.001). LD exposure had a clear effect on the time spent in the interaction zone (Kruskall–Wallis, H(5) = 42.38, p < 0.001) with 6 LD, 12 LD and 18 LD showing reduction compared to the basal group (Dunn’s multiple comparisons (p < 0.001) for each of the three comparisons. The number of interactions performed also was affected by LD exposure (one-way ANOVA, F(4,92) = 8.2, p < 0.001) with 6 LD, 12 LD, and 18 LD groups all performing fewer interactions compared to the Basal group [Dunnett’s multiple comparisons test (p < 0.001) for each of the three comparisons]. Download Figure 4-2, TIF file. [file enu-eN-NWR-0491-22-s09.tif]

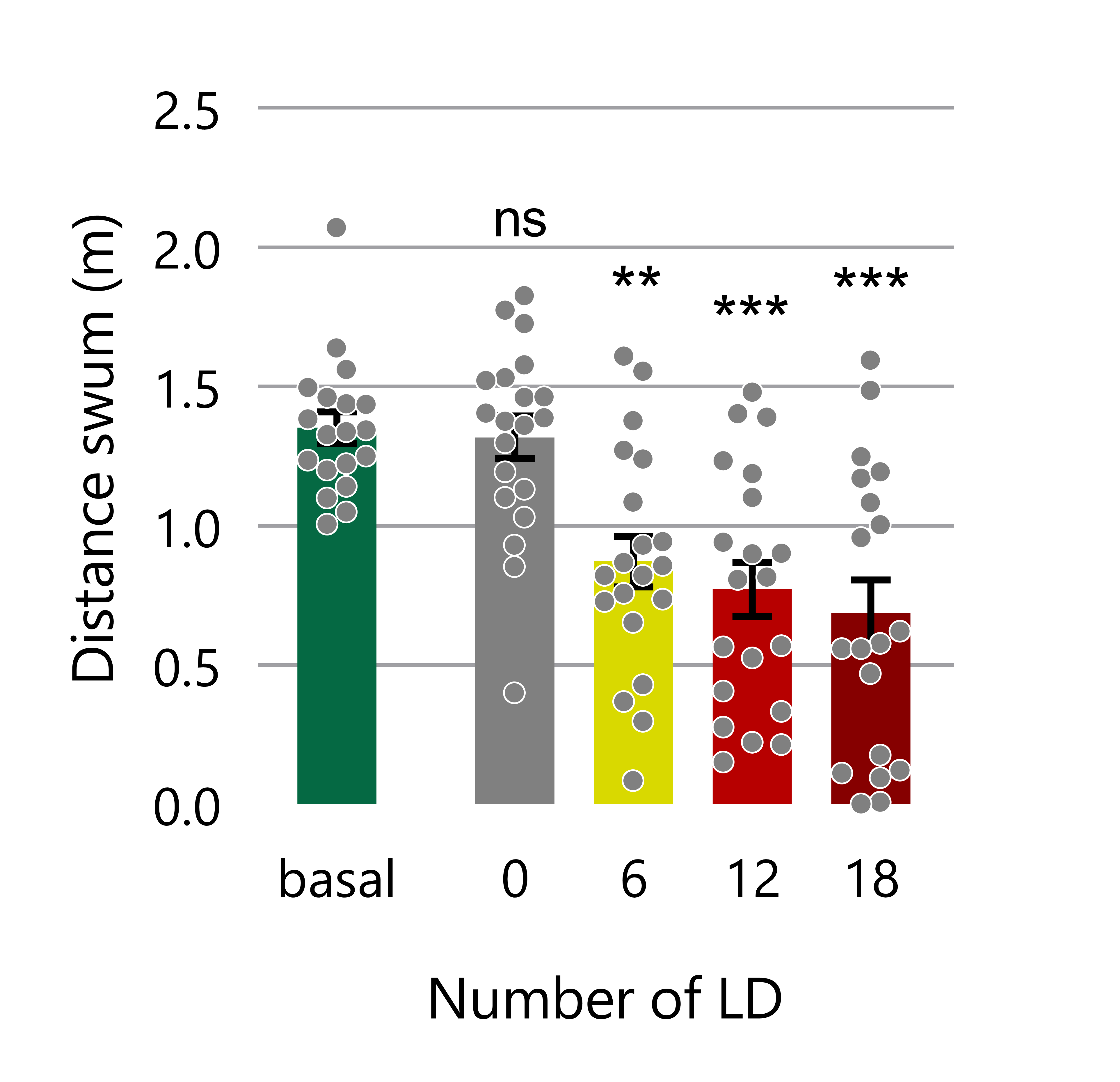

Supplement: Extended Data Figure 4-3 — Distance swum reduces in the social approach and maintenance following stress. Distance swum in meters in the social approach and maintenance assay following different intensities of stress exposure (ns p > 0.05; **p < 0.01, ***p < 0.001). Download Figure 4-3, TIF file. [file enu-eN-NWR-0491-22-s10.tif]
